# Risk cycling in diabetes and autism spectrum disorder: a bidirectional Mendelian randomization study

**DOI:** 10.3389/fendo.2024.1389947

**Published:** 2024-07-15

**Authors:** Yunfeng Yu, Xinyu Yang, Gang Hu, Keke Tong, Jingyi Wu, Rong Yu

**Affiliations:** ^1^ The First Hospital of Hunan University of Chinese Medicine, Changsha, Hunan, China; ^2^ School of Traditional Chinese Medicine, Hunan University of Chinese Medicine, Changsha, Hunan, China; ^3^ The Hospital of Hunan University of Traditional Chinese Medicine, Changde, Hunan, China; ^4^ The Third School of Clinical Medicine, Zhejiang Chinese Medical University, Hangzhou, Zhejiang, China

**Keywords:** type 1 diabetes mellitus, type 2 diabetes mellitus, gestational diabetes mellitus, autism spectrum disorder, bidirectional Mendelian randomization

## Abstract

**Objective:**

The relationship between diabetes mellitus (DM) and autism spectrum disorder (ASD) remains controversial. This study aimed to analyze the causal relationship between different types of DM and ASD by bidirectional Mendelian randomization (MR).

**Methods:**

Single nucleotide polymorphisms for type 1 diabetes mellitus (T1DM), type 2 diabetes mellitus (T2DM), gestational diabetes mellitus (GDM), and ASD were obtained from genome-wide association studies. Subsequently, inverse variance weighted, MR-Egger, and weighted median were used to test the exposure-outcome causality. Finally, MR-Egger’s intercept, Cochran’s Q, and leave-one-out method were used to assess horizontal pleiotropy, heterogeneity, and sensitivity of the results, respectively.

**Results:**

The positive analysis showed that T2DM was associated with an increased risk of ASD, whereas neither T1DM nor GDM was associated with the risk of ASD. The reverse analysis showed that ASD was associated with an increased risk of T2DM, while it was not associated with the risk of either T1DM or GDM. MR-Egger intercept showed no horizontal pleiotropy (*p* > 0.05) for these results. Cochran’s Q showed no heterogeneity expect for the results of T1DM on the risk of ASD, and leave-one-out sensitivity analysis showed these results were robust.

**Conclusion:**

This MR analysis suggests that T2DM and ASD are reciprocal risk factors and that they may create an intergenerational risk cycling in female patients. Aggressive prevention and treatment of T2DM and ASD help to break the trap of this risk cycling. Additionally, this study does not support a causal relationship between T1DM and ASD, as well as GDM and ASD. And more studies are needed in the future to continue to explore the interactions and underlying mechanisms between different types of DM and ASD.

## 1 Introduction

Autism spectrum disorder (ASD), caused by neurodevelopmental disorders, is characterized by impaired social communication, restricted interests, and repetitive behaviors (1). The pathogenesis of ASD has not yet been elucidated, but it is generally believed that oxidative stress and mitochondrial dysfunction cause synaptic and myelin dysfunction, leading to abnormal brain development (2). Epidemiological studies have shown that the global prevalence of ASD is about 1% and is increasing (3). Impaired social communication and interaction, restricted behavior and interests, as well as repetitive patterns are the main clinical manifestations of ASD (4), which seriously affect the health, education, and employment of patients (5). There is currently no effective treatment for ASD, and controlling major risk factors such as lifestyle during pregnancy, pre-pregnancy obesity, and stress is an effective means to reduce the risk of ASD in offspring (6). In recent years, more and more studies have found that diabetes mellitus (DM) is associated with the risk of ASD in offspring (7). The evidence points to the possibility that DM is an independent risk factor for ASD (8).

DM is an endocrine disease characterized by relative or absolute insulin deficiency due to dysfunction or destruction of pancreatic β-cells. Epidemiological research shows that there are nearly 415 million people with DM worldwide, and the incidence of DM is still increasing (9). Type 1 diabetes mellitus (T1DM) and type 2 diabetes mellitus (T2DM) are the two most common types of DM, with the former accounting for 90–95% of all DM cases and the latter accounting for 5–10% of all DM cases. T1DM is caused by absolute insulin deficiency due to the autoimmune destruction of pancreatic β-cells, and T2DM is caused by relative insulin deficiency due to insulin resistance. As a specific type of DM, gestational diabetes mellitus (GDM) occurs as a result of increased insulin resistance and impaired compensatory increase in insulin secretion during pregnancy, which is a metabolic disorder considered to occur in the pre-diabetic period. The persistent hyperglycemic state is a major cause of vascular and neuropathic disorders and is associated with a higher risk of cognitive impairment (10). As a typical neurodevelopmental disorder, ASD is thought to be associated with DM. Several studies have found that DM increases the risk of ASD in offspring (8, 11–14), and that ASD also increases their own risk of DM (15), which implies that there is an interaction between DM and ASD. However, other clinical reports have negated the causal relationship between DM and the risk of ASD (16). Whether there is an intrinsic link between ASD and DM remains a controversial topic, and the interaction between different types of DM and ASD has not been fully elucidated.

Mendelian randomization (MR), a means of analyzing the causal effect of exposure on outcomes through genetic variants, is an emerging method for epidemiological research (17). Since genotypes are randomly assigned, MR is unlikely to be affected by confounding variables, thus providing reliability and accuracy to its results (18). The study aims to analyze the causal relationship of ASD with different types of DM using MR analysis based on the large-scale genetic data and clinical information.

## 2 Materials and methods

### 2.1 Study design

**Figure 1:**
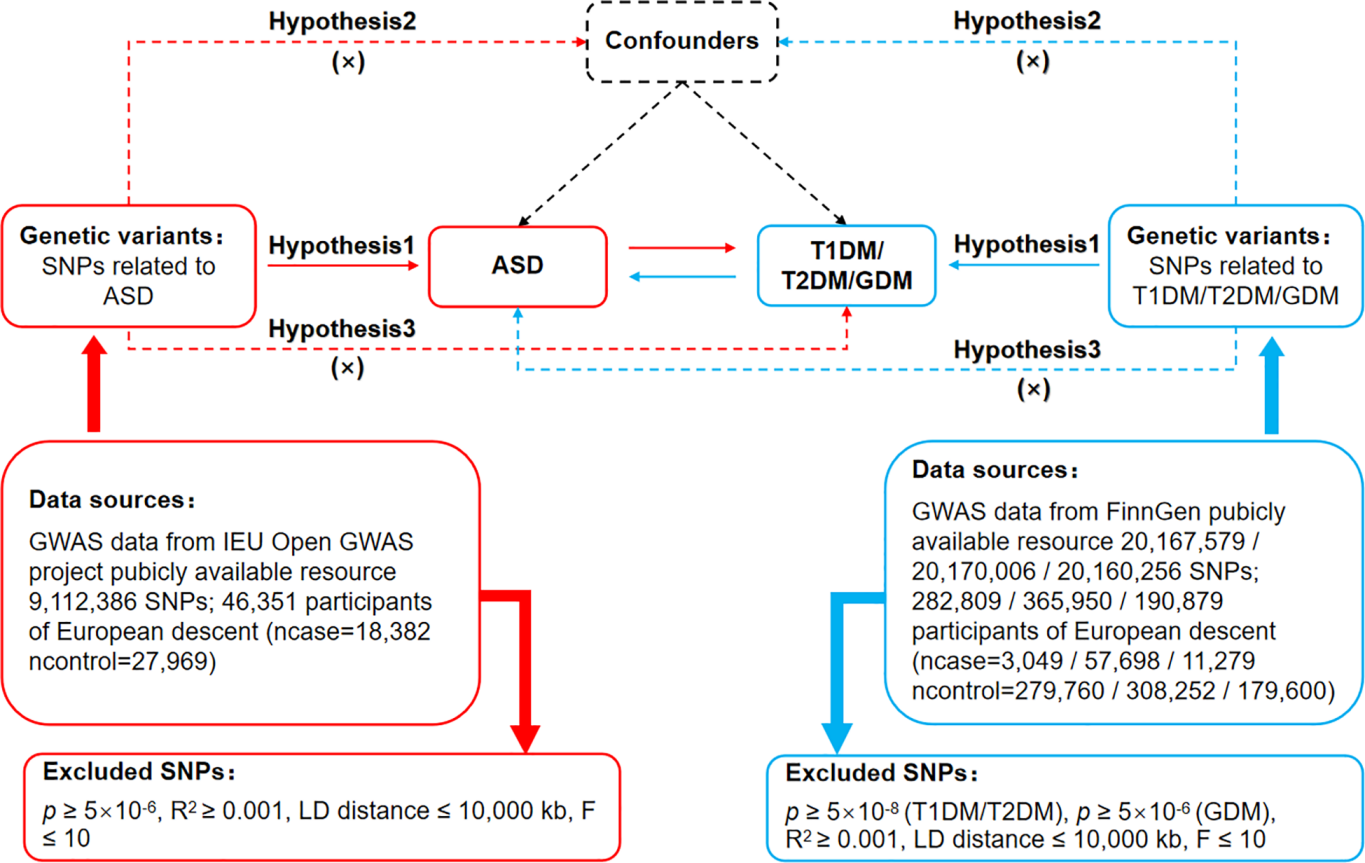
Bidirectional Mendelian randomization design for causal analysis of ASD and DM. ASD, autism spectrum disorder; DM, diabetes mellitus; T1DM, type 1 diabetes mellitus; T2DM, type 2 diabetes mellitus; GDM, gestational diabetes mellitus.

The MR’s protocol was based on three basic assumptions, as illustrated in **Figure 1**. The association assumption required that the single nucleotide polymorphism (SNP) was closely related to the exposure. The independence assumption required that the SNP was not influenced by confounding variables. The exclusivity assumption required that the SNP only acted on the outcome through the exposure and not through other pathways.

### 2.2 Data sources

The data for ASD was obtained from IEU open genome-wide association studies (GWAS) project (gwas.mrcieu.ac.uk/), and the data for T1DM, T2DM, and GDM were obtained from FinnGen (www.finngen.fi/fi), as shown in **Table 1**. The data for ASD contained 46,351 Europeans and 933 closely related SNPs, dataset number: ieu-a-1185. The data for T1DM contained 282,809 Europeans and 32,038 closely related SNPs, dataset number: finngen_R8_T1D_STRICT1. Data for T2DM contained 365,950 Europeans and 16,143 closely related SNPs, dataset number: finngen_R9_T2D. Data for GDM contained 190,879 Europeans and 2,087 closely related SNPs, dataset number: finngen_R8_GEST_DIABETES. As the databases are open, ethical approval is no longer required.

**Table 1:** Details of the GWAS studies included in the Mendelian randomization.

Year	Trait	GWAS ID	Population	Sample size	Web source
2017	ASD	ieu-a-1185	European	46,351	gwas.mrcieu.ac.uk/
2022	T1DM	finngen_R8_T1D_STRICT1	European	282,809	www.finngen.fi/fi
2023	T2DM	finngen_R9_T2D	European	365,950	www.finngen.fi/fi
2022	GDM	finngen_R8_GEST_DIABETES	European	190,879	www.finngen.fi/fi

ASD, autism spectrum disorder; T1DM, type 1 diabetes mellitus; T2DM, type 2 diabetes mellitus; GDM, gestational diabetes mellitus.

### 2.3 Selection of genetic instrument variables

Firstly, *p* < 5×10^-8^ was restricted to search for SNPs strongly linked to T1DM and T2DM, while *p* < 5×10^-6^ was restricted to search for SNPs strongly linked to ASD and GDM to fulfill the association assumption. Secondly, limiting kb = 10,000 and R^2^ < 0.001 to exclude the interference of linkage disequilibrium. Thirdly, SNPs with strong correlation were searched for with a threshold of F > 10, where F = [*R* ^2^/(1-*R* ^2^)]*[(*N-K*-1)/*k*], R^2^ representing the cumulative explained variance, N representing the sample size of the GWAS, and K representing the number of paired samples. Fourthly, SNPs with confounding variables were excluded by PhenoScanner and related literature to fulfill the independence assumption. Fifthly, the direction of exposure and outcome alleles were adjusted, and non-matching SNPs were excluded based on the effect allele frequency. Lastly, MR-Pleiotropy RESidual Sum and Outlier (MR-PRESSO) was used to eliminate outlier SNPs (*p* < 1) to ensure the correct causal inference.

### 2.4 Data analysis

The STROBE-MR was used as a guiding method (19). Two-sample bidirectional MR was used to assess the causal relationship of ASD with T1DM, T2DM, and GDM. The “TwoSampleMR (0.5.7)” package of R 4.3.1 was used to perform all operations of the MR analysis. IVW was set as the primary assessment tool, which achieved unbiased causal analysis in the absence of pleiotropy. Weighted median, which was sensitive to outliers, and MR-Egger, which analyzed data in the presence of pleiotropy, were set as secondary assessment tools. The MR-Egger’s intercept was also used to assess horizontal pleiotropy, which was required to satisfy the exclusivity assumption (*p* ≥ 0.05). Cochran’s Q and leave-one-out method were used to analyze heterogeneity and sensitivity, respectively. There was no heterogeneity in the results at *p* ≥ 0.05, and the results were robust when there were no significant changes in the combined effect sizes.

## 3 Results

### 3.1 Genetic instrument variables

After association, independence and exclusivity tests, this study included 32 SNPs for ASD, 21 SNPs for T1DM, 148 SNPs for T2DM, and 38 SNPs for GDM (**Supplementary Table S1**). Then, mismatched and outlier SNPs were excluded based on effect allele frequency and MR-PRESSO, respectively. **Supplementary Table S2** illustrates the final included SNPs.

### 3.2 Bidirectional MR analysis

**Figure 2:**
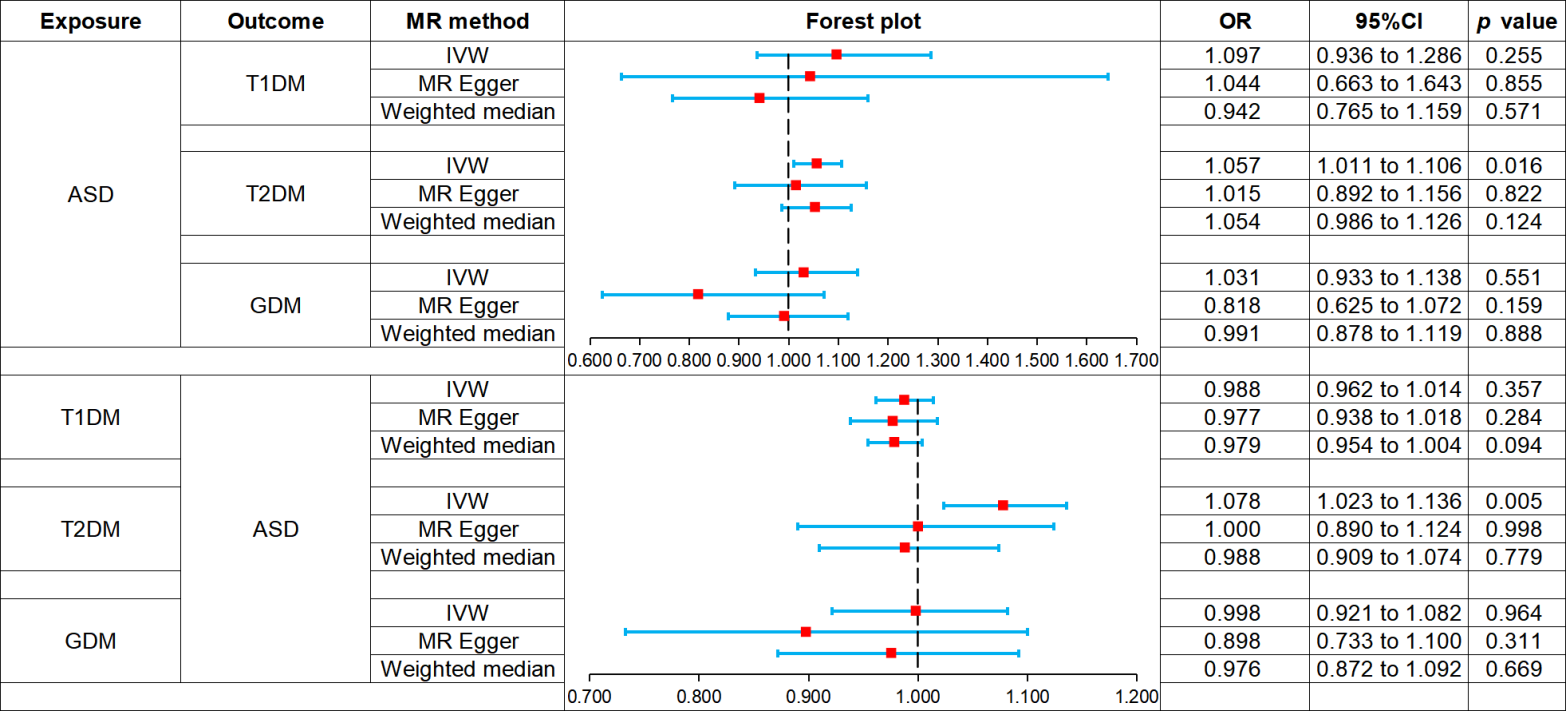
Forest plot of Mendelian randomization analysis on the causal relationship between ASD and DM. ASD, autism spectrum disorder; DM, diabetes mellitus; T1DM, type 1 diabetes mellitus; T2DM, type 2 diabetes mellitus; GDM, gestational diabetes mellitus.

**Figure 3:**
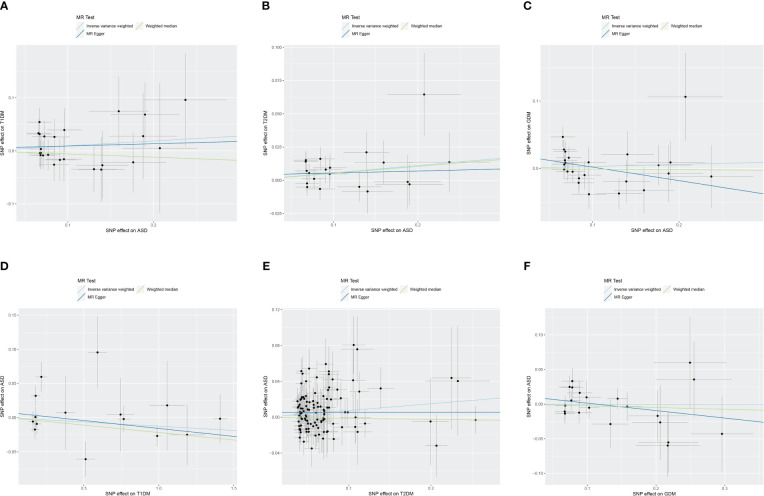
Scatter plot of Mendelian randomization analysis on the causal relationship between ASD and DM. **(A)** ASD on T1DM; **(B)** ASD on T2DM; **(C)** ASD on GDM; **(D)** T1DM on ASD; **(E)** T2DM on ASD; **(F)** GDM on ASD. ASD, autism spectrum disorder; DM, diabetes mellitus; T1DM, type 1 diabetes mellitus; T2DM, type 2 diabetes mellitus; GDM, gestational diabetes mellitus.

Two-sample bidirectional MR analysis assessed the causal effect between ASD and DM. **Figure 2** presents the forest plot, while **Figure 3** displays a scatter plot. The MR-Egger’s intercept assessed the horizontal pleiotropy of results, as shown in **Supplementary Table S3**.

#### 3.2.1 The effect of ASD on T1DM

MR analysis showed that ASD was not associated with the risk of T1DM: IVW (odds ratio [OR] 1.097, 95% confidence interval [CI] 0.936 to 1.286, *p* = 0.255), MR-Egger (OR 1.044, 95% CI 0.663 to 1.643, *p* = 0.855), weighted median (OR 0.942, 95% CI 0.765 to 1.159, *p* = 0.571). And there was no horizontal pleiotropy in the results (*p* = 0.821).

#### 3.2.2 The effect of ASD on T2DM

IVW (OR 1.057, 95% CI 1.011 to 1.106, *p* = 0.016) showed that ASD was associated with increased risk of T2DM, while MR-Egger (OR 1.015, 95% CI 0.892 to 1.156, *p* = 0.822) and weighted median (OR 1.054, 95% CI 0.986 to 1.126, *p* = 0.124) did not observe this causal relationship. And there was no horizontal pleiotropy in the results (*p* = 0.524).

#### 3.2.3 The effect of ASD on GDM

MR analysis showed that ASD was not associated with the risk of GDM: IVW (OR 1.031, 95% CI 0.933 to 1.138, *p* = 0.551), MR-Egger (OR 0.818, 95% CI 0.625 to 1.072, *p* = 0.159), weighted median (OR 0.991, 95% CI 0.878 to 1.119, *p* = 0.888). And there was no horizontal pleiotropy in the results (*p* = 0.087).

#### 3.2.4 The effect of T1DM on ASD

MR analysis showed that T1DM was not associated with the risk of ASD: IVW (OR 0.988, 95% CI 0.962 to 1.014, *p* = 0.357), MR-Egger (OR 0.977, 95% CI 0.938 to 1.018, *p* = 0.284), weighted median (OR 0.979, 95% CI 0.954 to 1.004, *p* = 0.094). And there was no horizontal pleiotropy in the results (*p* = 0.501).

#### 3.2.5 The effect of T2DM on ASD

IVW (OR 1.078, 95% CI 1.023 to 1.136, *p* = 0.005) showed that T2DM was associated with increased the risk of ASD, while MR-Egger (OR 1.000, 95% CI 0.890 to 1.124, *p* = 0.998) and weighted median (OR 0.988, 95% CI 0.909 to 1.074, *p* = 0.779) did not observe this causal relationship. And there was no horizontal pleiotropy in the results (*p* = 0.164).

#### 3.2.6 The effect of GDM on ASD

MR analysis showed that GDM was not associated with the risk of ASD: IVW (OR 0.998, 95% CI 0.921 to 1.082, *p* = 0.964), MR-Egger (OR 0.898, 95% CI 0.733 to 1.100, *p* = 0.311), weighted median (OR 0.976, 95% CI 0.872 to 1.092, *p* = 0.669). And there was no horizontal pleiotropy in the results (*p* = 0.279).

### 3.3 Heterogeneity and sensitivity analysis

**Figure 4:**
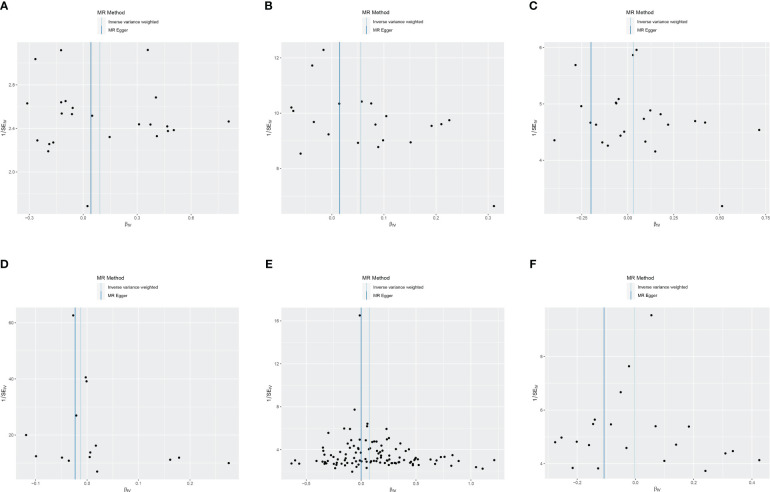
Funnel plot of heterogeneity analysis on ASD and DM. **(A)** ASD on T1DM; **(B)** ASD on T2DM; **(C)** ASD on GDM; **(D)** T1DM on ASD; **(E)** T2DM on ASD; **(F)** GDM on ASD. ASD, autism spectrum disorder; DM, diabetes mellitus; T1DM, type 1 diabetes mellitus; T2DM, type 2 diabetes mellitus; GDM, gestational diabetes mellitus.

**Figure 5:**
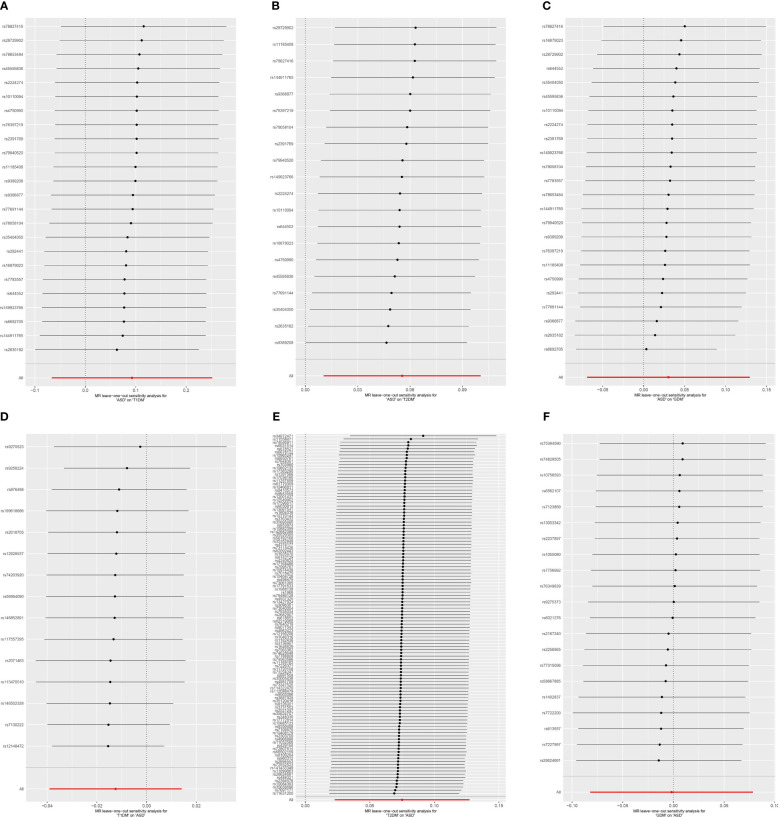
Leave-one-out sensitivity analysis on ASD and DM. **(A)** ASD on T1DM; **(B)** ASD on T2DM; **(C)** ASD on GDM; **(D)** T1DM on ASD; **(E)** T2DM on ASD; **(F)** GDM on ASD. ASD, autism spectrum disorder; DM, diabetes mellitus; T1DM, type 1 diabetes mellitus; T2DM, type 2 diabetes mellitus; GDM, gestational diabetes mellitus.

Cochran’s Q revealed heterogeneity only in the results of T1DM on the risk of ASD (*p* = 0.036), while the remaining results showed no significant heterogeneity (*p* ≥ 0.05), as depicted in **Figure 4** and **Supplementary Table S4**. The illustrated sensitivity analysis affirmed the robustness of all MR analysis results in **Figure 5**.

## 4 Discussion

### 4.1 Background and purpose

As a growing public health problem, ASD increases the risk of suicide and self-harm in patients and brings a significant burden on families and society (20). Early detection and intervention are essential to prevent behavioral deficits and complications of ASD (21). Parental illness, gestational hypertension, and preterm delivery are considered major risk factors for ASD (22, 23), and controlling these risk factors helps to reduce the risk of ASD. As research has progressed, some researchers have found that maternal DM is associated with an increased risk of ASD in offspring (11, 12), and that ASD increases the risk of DM in the same individual (15). However, other studies have shown that DM has no significant effect on the incidence of ASD (16). The causal relationship between ASD and different types of DM is controversial. Given the controversial causal relationship between ASD and different types of DM, we used MR to evaluate the bidirectional relationship between them. This is the first MR study based on GWAS data that comprehensively analyzes the bidirectional causal relationship between three types of DM and ASD, ultimately revealing the risk cycling between T2DM and ASD.

### 4.2 Analysis of results

This MR analysis showed that ASD and T2DM were associated with an increased risk of each other in European populations, whereas there was no causal relationship between ASD and T1DM or between ASD and GDM. Intercept analysis showed no horizontal pleiotropy for any of these outcomes. Cochran’s Q test showed no heterogeneity for any of the outcomes except for exposure (T1D)-outcome (ASD). Since the entire dataset was derived from Europeans, this study mainly explained the interaction of ASD with T1DM, T2DM, and GDM in Europeans.

### 4.3 Impact of T2DM on ASD

Our study finds that T2DM is associated with an increased risk of ASD. A published clinical study supports this result, suggesting that T2DM is an independent risk factor for ASD in offspring (24). A cohort study that included 877,233 singleton infants born in Taiwan found that T2DM was associated with an increased risk of neurodevelopmental disorders (NDDs) such as ASD, attention deficit hyperactivity disorder (ADHD), developmental delays, and intellectual disabilities (25). A cohort study by the Swedish Psychiatric Association showed that pre-pregnancy T2DM was associated with an increased incidence of NDDs in offspring and that it increased the risk of ASD by 37% and ADHD by 43% (26). Yamamoto JM et al.’s (8) meta-analysis showed that T2DM exposure during pregnancy increased the risk of ASD in offspring by approximately 2-fold (OR 1.98, 95% CI 1.46 to 2.68). These pieces of evidence suggest that maternal T2DM is a potential risk factor for ASD in offspring, and a persistent hyperglycemic state may mediate this causal relationship (25). For one thing, hyperglycemia causes intrauterine hypoxia, resulting in impaired fetal neurodevelopment; for another thing, hyperglycemia induces free radical production and oxidative stress, which can affect fetal neurodevelopment (27). These suggest that hypoxia and oxidative stress mediated by hyperglycemia may be the mechanism by which T2DM increases the risk of ASD.

### 4.4 Impact of GDM on ASD

This MR analysis suggests that GDM is not associated with the risk of ASD, a result supported by several clinical studies. A US cohort study showed that the risk of ASD in offspring of patients with GDM diagnosed at 26 weeks of gestation and later was comparable to that of subjects without DM exposure (OR 0.99, 95% CI 0.88 to 1.12) (28). Another Spanish study that included 1,037 GDM pregnant women with singleton showed that GDM was associated with an increased risk of ADHD in offspring but not with the risk of ASD (29). Moussa HN et al.’s (30) systematic review pointed out that factors such as GDM, chronic hypertension, and autoimmune diseases were not associated with the progression of ASD in offspring. These pieces of evidence point to GDM not being a risk factor for ASD in offspring.

However, more studies support that GDM increases the risk of ASD in offspring. A US cohort study showed that pregnant women diagnosed with GDM at 26 weeks of gestation had a 63% increased incidence of ASD in their offspring (HR 1.63, 95% CI 1.35 to 1.97) (31). A meta-analysis that included 17 clinical studies showed that the offspring of patients with GDM had a 49% increased risk of developing ASD compared to non-diabetic patients (OR 1.49, 95% CI 1.18 to 1.89) (32). These studies support GDM as an independent risk factor for ASD in offspring, in contrast to this MR result.

Several reasons are used to explain the difference in results between MR analysis and clinical studies. First, the effect of GDM on ASD is related to the time of onset. Xiang AH et al.’s (28) cohort study showed that GDM diagnosed before 26 weeks of gestation resulted in a 30% additional risk of ASD, whereas GDM diagnosed after 26 weeks of gestation was not associated with the risk of ASD. In fact, the period before 24 weeks is the critical stage of fetal brain and spinal cord development (33). This means that the earlier the hyperglycemia occurs, the more lasting and significant the neurodevelopmental effects are (33). Unfortunately, this MR only analyzed the effect of the presence or absence of GDM on the risk of ASD and not the effect of GDM occurring at different gestational periods on the risk of ASD, as all the data in this study were derived from pooled GWAS data. Second, patients with GDM may include a subset of patients with impaired glucose tolerance or DM that was not diagnosed in a timely manner prior to pregnancy. In clinical trials, the additional risk of ASD in offspring of these patients was thought to be associated with GDM, but in fact, they were affected by T2DM rather than GDM. Third, considering that obesity is associated with lower insulin sensitivity and higher risk of GDM (34), the increased risk of ASD in offspring observed in clinical studies may be mediated by obesity rather than GDM. Kong L et al. (35) confirmed that the offspring of obese patients were at significantly increased risk of ASD, ADHD, and mixed disorder of conduct and emotion, but the risk of these disorders was not associated with GDM. In addition, three published MR studies support our speculation. Among them, the MR study by Ding H et al. (36) showed a correlation between obesity and increased genetic susceptibility to ASD, while the MR studies by Hu M et al. (37) and Song X et al. (38) supported a correlation between obesity and increased genetic susceptibility to GDM.

### 4.5 Impact of T1DM on ASD

Literature on the causal relationship between T1DM and ASD is scarce, and they provide some controversial results. A Colorado case-control study found that the prevalence of ASD in the pediatric T1DM population was approximately 1.16%, which is comparable to the general population in Colorado (39). Another retrospective cohort study in Taiwan showed that T1DM exposure was associated with an increased risk of developmental delay, intellectual disability, and epilepsy in offspring but not with the risk of ASD (25). These pieces of evidence point to no significant effect of T1DM on either one’s own risk of ASD or the risk of ASD in offspring, consistent with the results of this MR analysis.

However, Chen MH et al. (40), in a Cox regression analysis of data from the Taiwan National Health Insurance Research Database, found a significant increase in ASD prevalence in patients with T1DM compared with controls. In a California retrospective cohort study, Xiang AH et al. (28) reported that T1DM was still associated with an increased risk of ASD in offspring after adjusting for smoking and pre-pregnancy body mass index (HR 2.33, 95% CI 1.29 to 4.21). Although these two studies support T1DM as a potential risk factor for ASD in offspring, they included different population than our MR analysis. The participants of the first study were Chinese belonging to Asian ethnicity, and the participants of the second study were Americans who included a multiracial population of European, Latino, African, and Asian ethnicity, whereas the subjects of our MR study were of European ethnicity. These racial disparities could potentially explain the variations in findings between the clinical study and our MR analysis. Additionally, Persson M et al. (23) found that maternal T1DM increased the risk of ASD by 40% in offspring in a Swedish population. According to our speculation, the effect of T1DM on the risk of ASD in offspring is mediated through preterm births. This is because T1DM leads to a higher risk of preterm births (28), and preterm births increase the risk of ASD in turn (41). The presence of an intermediate link, preterm births, may account for the difference between the MR analysis and clinical studies. In summary, considering the current lack of clinical evidence, more clinical studies are needed in the future to explore the potential role of T1DM in ASD.

### 4.6 Effect of ASD on DM

In the reverse analysis, our MR shows that ASD is associated with an increased risk of T2DM, with no significant effect on the risk of T1DM and GDM. Although the effect of ASD on the risk of GDM has not been reported, current studies support the results of MR analysis of T1DM and T2DM. Zerbo O et al.’s (42) case-control study showed comparable rates of T1DM diagnosis in children with and without ASD (0.22% vs. 0.19%), supporting that ASD is not associated with the risk of T1DM. Chen MH and his colleagues (43) conducted a clinical study in Taiwan that showed both adolescents (HR 2.71, 95% CI 1.64 to 4.48) and young adults (HR 5.31, 95% CI 2.85 to 9.90) with ASD had a significantly increased risk of T2DM compared with age-matched controls. Even with short-term (HR 1.97, 95% CI 1.20 to 3.23) or long-term (HR 1.64, 95% CI 1.02 to 2.63) use of atypical antipsychotic medications, patients with ASD continued to have a higher risk of T2DM (43). Chieh AY et al.’s (15) systematic review still supports ASD as a potential risk factor for T2DM and metabolic disorders after excluding the interference of some confounding variables. These show that ASD is a potential risk factor for T2DM rather than T1DM.

In conclusion, ASD and T2DM are risk factors for each other, that T2DM increases the risk of ASD in offspring, and that ASD in turn increases its own risk of T2DM, that they may create an intergenerational risk cycling in female patients. Aggressive prevention and treatment of T2DM and ASD help to break the trap of this risk cycling.

### 4.7 Reports on related SNPs

To further elucidate the genetic mechanisms underlying the interaction between ASD and T2DM, we conducted a search in public databases for 20 SNPs for ASD acting on T2DM and 113 SNPs for T2DM acting on ASD. Among them, 13 SNPs have relevant literature reports. The rs3887925, rs11558471, rs6931514, rs34872471, rs6878122, rs7018475, rs348330, rs4862423, and rs878521 have been reported to be related to glucose metabolism or diabetes, while the rest of SNPs have not been reported to be related to T2DM. All SNPs have not been reported to be related to ASD. As shown in **Supplementary Table S5**.

### 4.8 Limitations and prospects

While this MR analysis enhances the genetic evidence, there are inevitably some limitations. First, as the data were derived from Europeans, our findings may not extrapolate to elucidate the relationship of ASD and DM in other racial groups. Second, since GWAS does not provide specific information on when GDM was diagnosed, this MR analysis can only explain changes in the risk of ASD at the time of GDM exposure and cannot analyze the effect of GDM occurring at different gestational periods on the risk of ASD. Third, obesity may be intrinsic to the increased risk of GDM in oneself and the risk of ASD in offspring, but no sufficient clinical evidence has yet supported this inference. Fourth, the effect of T1DM in increasing the risk of ASD may be mediated through preterm birth, but no high-quality clinical study has yet analyzed this scenario. Fifth, there may exist unrecognized confounding variables between exposure factors and outcome indicators, which increase the risk of bias in MR results.

Acknowledging these limitations, we anticipate that future researchers will continue to enrich the GWAS database, aiming for racially diverse MR research and promoting health equity. Second, stratified clinical studies should be conducted to explore the effect of GDM at different gestational periods on the risk of ASD in offspring. Third, researchers are expected to use large sample and multicenter clinical studies to analyze the role of obesity on the risk of GDM and ASD, as well as the role of T1DM on the risk of preterm birth and preterm birth on the risk of ASD, to validate the speculations of the study further.

## 5 Conclusion

This MR analysis suggests that T2DM and ASD are reciprocal risk factors. T2DM increases the risk of ASD in offspring, while ASD increases one’s own risk of T2DM, and that they may create an intergenerational risk cycling in female patients. Aggressive prevention and treatment of T2DM and ASD help to break the trap of this risk cycling. Additionally, this study does not support a causal relationship between T1DM and ASD, as well as GDM and ASD, and more research is needed to explore this topic in the future.

## Data availability statement

The original contributions presented in the study are included in the article/**Supplementary Material**. Further inquiries can be directed to the corresponding author.

## Author contributions

YY: Conceptualization, Data curation, Supervision, Writing – original draft. XY: Formal Analysis, Methodology, Writing – original draft. GH: Data curation, Methodology, Writing – original draft. KT: Data curation, Formal Analysis, Writing – original draft. JW: Data curation, Writing – original draft. RY: Supervision, Writing – review & editing.
