# Supplementary material for: Risk cycling in diabetes and autism spectrum disorder: a bidirectional Mendelian randomization study
Source: Front Endocrinol (Lausanne). 2024 Jul 15;15:1389947. doi: 10.3389/fendo.2024.1389947 (PMC11328693; doi:10.3389/fendo.2024.1389947)
Supplement: Supplementary file 1 [file Table_1.docx]

**Supplementary Table S1.1** Characteristics of instrumental variables for ASD.

|  | **SNP** | **EA** | **OA** | **Samplesize** | **SE** | **β** | **id.exposure** | **EAF** | ***p* value** | **R^2^** | **F - statistic** |
| --- | --- | --- | --- | --- | --- | --- | --- | --- | --- | --- | --- |
| 1 | rs2391769 | G | A | 46,351 | 0.0145 | 0.0769026 | ieu-a-1185 | 0.33 | 1.14E-07 | 0.002615175 | 121.5285725 |
| 2 | rs6701243 | C | A | 46,351 | 0.0144 | -0.0735014 | ieu-a-1185 | 0.6412 | 3.32E-07 | 0.002485806 | 115.5017197 |
| 3 | rs11185408 | A | G | 46,351 | 0.0138 | -0.0686965 | ieu-a-1185 | 0.5089 | 6.42E-07 | 0.002358857 | 109.5891654 |
| 4 | rs78653484 | T | C | 46,351 | 0.0385 | -0.176296 | ieu-a-1185 | 0.9493 | 4.67E-06 | 0.002991757 | 139.0810539 |
| 5 | rs6692705 | G | A | 46,351 | 0.0141 | -0.0656005 | ieu-a-1185 | 0.3907 | 3.28E-06 | 0.002048891 | 95.15902586 |
| 6 | rs79940520 | G | A | 46,351 | 0.0207 | 0.0953992 | ieu-a-1185 | 0.8519 | 4.05E-06 | 0.002296482 | 106.6846663 |
| 7 | rs2635182 | T | C | 46,351 | 0.014 | 0.0669014 | ieu-a-1185 | 0.5278 | 1.76E-06 | 0.002230981 | 103.6349232 |
| 8 | rs9366877 | G | A | 46,351 | 0.0139 | -0.0684994 | ieu-a-1185 | 0.5726 | 8.31E-07 | 0.002296621 | 106.6911307 |
| 9 | rs16879023 | A | G | 46,351 | 0.0201 | -0.0957953 | ieu-a-1185 | 0.1521 | 1.88E-06 | 0.002366967 | 109.9668529 |
| 10 | rs12203328 | C | G | 46,351 | 0.0153 | 0.0697033 | ieu-a-1185 | 0.2445 | 5.22E-06 | 0.00179494 | 83.34328391 |
| 11 | rs740883 | T | A | 46,351 | 0.0238 | 0.113695 | ieu-a-1185 | 0.0974 | 1.78E-06 | 0.00227283 | 105.5833855 |
| 12 | rs72934503 | G | A | 46,351 | 0.0141 | 0.0704976 | ieu-a-1185 | 0.5169 | 5.74E-07 | 0.002482117 | 115.329898 |
| 13 | rs9389208 | T | C | 46,351 | 0.0144 | 0.0672006 | ieu-a-1185 | 0.6233 | 3.06E-06 | 0.00212065 | 98.49889926 |
| 14 | rs7783557 | C | T | 46,351 | 0.0146 | -0.0670042 | ieu-a-1185 | 0.3211 | 4.45E-06 | 0.001957403 | 90.90158444 |
| 15 | rs111931861 | G | A | 46,351 | 0.0409 | 0.216901 | ieu-a-1185 | 0.9433 | 1.14E-07 | 0.005032526 | 234.4323183 |
| 16 | rs10099100 | C | G | 46,351 | 0.0147 | 0.0843044 | ieu-a-1185 | 0.341 | 9.75E-09 | 0.00319426 | 148.5251882 |
| 17 | rs76397219 | G | A | 46,351 | 0.0303 | 0.140297 | ieu-a-1185 | 0.9175 | 3.65E-06 | 0.002979798 | 138.5234171 |
| 18 | rs10110094 | G | A | 46,351 | 0.0191 | -0.0906996 | ieu-a-1185 | 0.1451 | 2.05E-06 | 0.002040908 | 94.78750634 |
| 19 | rs28729902 | G | A | 46,351 | 0.0178 | 0.0839035 | ieu-a-1185 | 0.8042 | 2.43E-06 | 0.002217006 | 102.9843372 |
| 20 | rs45595836 | T | C | 46,351 | 0.0272 | 0.138996 | ieu-a-1185 | 0.9066 | 3.22E-07 | 0.003271879 | 152.1461084 |
| 21 | rs141319505 | G | A | 46,351 | 0.061 | -0.290698 | ieu-a-1185 | 0.9692 | 1.88E-06 | 0.005045198 | 235.0256277 |
| 22 | rs78827416 | A | G | 46,351 | 0.0266 | 0.130502 | ieu-a-1185 | 0.0746 | 9.29E-07 | 0.002351433 | 109.243458 |
| 23 | rs4750990 | C | T | 46,351 | 0.0141 | 0.0680968 | ieu-a-1185 | 0.3857 | 1.37E-06 | 0.002197422 | 102.07263 |
| 24 | rs644552 | A | G | 46,351 | 0.0346 | 0.159403 | ieu-a-1185 | 0.0626 | 4.08E-06 | 0.0029821 | 138.6307818 |
| 25 | rs35404050 | T | C | 46,351 | 0.0176 | 0.0843044 | ieu-a-1185 | 0.7883 | 1.67E-06 | 0.002372154 | 110.208393 |
| 26 | rs77691144 | C | T | 46,351 | 0.0435 | 0.207406 | ieu-a-1185 | 0.0318 | 1.86E-06 | 0.002648896 | 123.0997359 |
| 27 | rs78058104 | A | G | 46,351 | 0.0397 | 0.187898 | ieu-a-1185 | 0.0328 | 2.21E-06 | 0.002240085 | 104.0587868 |
| 28 | rs141455452 | G | T | 46,351 | 0.0159 | -0.0784044 | ieu-a-1185 | 0.4334 | 8.18E-07 | 0.003019092 | 140.3556407 |
| 29 | rs292441 | A | G | 46,351 | 0.0149 | -0.0724954 | ieu-a-1185 | 0.327 | 1.14E-06 | 0.002313203 | 107.4632218 |
| 30 | rs149923766 | G | T | 46,351 | 0.0484 | 0.237306 | ieu-a-1185 | 0.0119 | 9.44E-07 | 0.001324327 | 61.46263737 |
| 31 | rs2224274 | T | C | 46,351 | 0.0138 | 0.0709989 | ieu-a-1185 | 0.498 | 2.68E-07 | 0.002520382 | 117.1123333 |
| 32 | rs144911765 | C | T | 46,351 | 0.0403 | 0.190096 | ieu-a-1185 | 0.0268 | 2.39E-06 | 0.001885006 | 87.53316583 |

SNP, single nucleotide polymorphism; EA, effect allele; OA, other allele; EAF, effect allele frequency; SE, standard error; ASD, autism spectrum disorder.

**Supplementary Table S1.2** Characteristics of instrumental variables for T1DM.

|  | **SNP** | **EA** | **OA** | **Samplesize** | **SE** | **β** | **id.exposure** | **EAF** | ***p* value** | **R^2^** | **F - statistic** |
| --- | --- | --- | --- | --- | --- | --- | --- | --- | --- | --- | --- |
| 1 | rs146502328 | A | G | 282,809 | 0.0634101 | 0.591597 | finngen_R8_T1D_STRICT1 | 0.0292366 | 1.06E-20 | 0.019866538 | 5732.276359 |
| 2 | rs140561556 | A | C | 282,809 | 0.199578 | 1.19611 | finngen_R8_T1D_STRICT1 | 0.00181858 | 2.06E-09 | 0.005194146 | 1476.610502 |
| 3 | rs9258224 | A | G | 282,809 | 0.0629088 | -0.51222 | finngen_R8_T1D_STRICT1 | 0.0605616 | 3.88E-16 | 0.029854425 | 8702.859165 |
| 4 | rs115470510 | A | G | 282,809 | 0.0460911 | 1.40903 | finngen_R8_T1D_STRICT1 | 0.0329285 | 1.00E-200 | 0.12644481 | 40935.56738 |
| 5 | rs146852891 | T | G | 282,809 | 0.0903046 | -0.745272 | finngen_R8_T1D_STRICT1 | 0.0340325 | 1.55E-16 | 0.036518756 | 10719.21209 |
| 6 | rs62404834 | A | T | 282,809 | 0.0733963 | -1.23239 | finngen_R8_T1D_STRICT1 | 0.0622245 | 2.85E-63 | 0.177250155 | 60926.88447 |
| 7 | rs9270523 | G | T | 282,809 | 0.0283344 | 0.98982 | finngen_R8_T1D_STRICT1 | 0.664683 | 1.00E-200 | 0.43672956 | 219273.3155 |
| 8 | rs2071463 | T | C | 282,809 | 0.0418953 | -0.766291 | finngen_R8_T1D_STRICT1 | 0.150275 | 9.83E-75 | 0.149962468 | 49892.42704 |
| 9 | rs117557295 | C | A | 282,809 | 0.117338 | -1.05621 | finngen_R8_T1D_STRICT1 | 0.0230121 | 2.23E-19 | 0.050162132 | 14935.39311 |
| 10 | rs199618686 | G | A | 282,809 | 0.121516 | -1.18908 | finngen_R8_T1D_STRICT1 | 0.0235513 | 1.30E-22 | 0.065030405 | 19670.21595 |
| 11 | rs6903171 | C | G | 282,809 | 0.0343861 | 0.636159 | finngen_R8_T1D_STRICT1 | 0.116026 | 2.05E-76 | 0.083014921 | 25602.59844 |
| 12 | rs2018705 | G | T | 282,809 | 0.0323633 | -0.186627 | finngen_R8_T1D_STRICT1 | 0.230959 | 8.09E-09 | 0.012372667 | 3542.912154 |
| 13 | rs7130222 | G | T | 282,809 | 0.0310595 | -0.179019 | finngen_R8_T1D_STRICT1 | 0.26203 | 8.23E-09 | 0.012394185 | 3549.151105 |
| 14 | rs689 | T | A | 282,809 | 0.0360737 | 0.643875 | finngen_R8_T1D_STRICT1 | 0.789634 | 2.95E-71 | 0.137731891 | 45173.35446 |
| 15 | rs56994090 | C | T | 282,809 | 0.0265483 | -0.177446 | finngen_R8_T1D_STRICT1 | 0.494549 | 2.33E-11 | 0.01574167 | 4523.054988 |
| 16 | rs12148472 | C | T | 282,809 | 0.0395748 | -0.216958 | finngen_R8_T1D_STRICT1 | 0.145436 | 4.20E-08 | 0.011700323 | 3348.107158 |
| 17 | rs12928537 | A | G | 282,809 | 0.0293578 | -0.16057 | finngen_R8_T1D_STRICT1 | 0.302464 | 4.51E-08 | 0.010879254 | 3110.569951 |
| 18 | rs55993634 | G | C | 282,809 | 0.0437082 | 0.258417 | finngen_R8_T1D_STRICT1 | 0.0886611 | 3.37E-09 | 0.010791584 | 3085.229903 |
| 19 | rs876498 | A | G | 282,809 | 0.0275611 | 0.17432 | finngen_R8_T1D_STRICT1 | 0.343741 | 2.53E-10 | 0.013709797 | 3931.121569 |
| 20 | rs74203920 | T | C | 282,809 | 0.0634671 | 0.377671 | finngen_R8_T1D_STRICT1 | 0.0366716 | 2.67E-09 | 0.010077702 | 2879.058853 |
| 21 | rs2074706 | C | G | 282,809 | 0.0274726 | 0.181612 | finngen_R8_T1D_STRICT1 | 0.336424 | 3.83E-11 | 0.014726404 | 4226.978402 |

SNP, single nucleotide polymorphism; EA, effect allele; OA, other allele; EAF, effect allele frequency; SE, standard error; T1D, type 1 diabetes mellitus.

**Supplementary Table S1.3** Characteristics of instrumental variables for T2DM.

|  | **SNP** | **EA** | **OA** | **Samplesize** | **SE** | **β** | **id.exposure** | **EAF** | ***p* value** | **R^2^** | **F - statistic** |
| --- | --- | --- | --- | --- | --- | --- | --- | --- | --- | --- | --- |
| 1 | rs1925670 | G | T | 365,950 | 0.00684577 | -0.0390442 | finngen_R9_T2D | 0.524394 | 1.17E-08 | 0.00076041 | 278.4824542 |
| 2 | rs61779309 | C | T | 365,950 | 0.00861286 | 0.0629103 | finngen_R9_T2D | 0.19256 | 2.79E-13 | 0.001230693 | 450.9247115 |
| 3 | rs17383290 | G | A | 365,950 | 0.00773841 | -0.0484758 | finngen_R9_T2D | 0.268289 | 3.74E-10 | 0.000922619 | 337.9423872 |
| 4 | rs3736907 | A | C | 365,950 | 0.00772418 | -0.0439305 | finngen_R9_T2D | 0.267554 | 1.29E-08 | 0.000756396 | 277.011213 |
| 5 | rs10802132 | T | C | 365,950 | 0.00724107 | 0.0404602 | finngen_R9_T2D | 0.323223 | 2.30E-08 | 0.000716199 | 262.2795469 |
| 6 | rs112088479 | T | C | 365,950 | 0.00988469 | 0.069229 | finngen_R9_T2D | 0.137826 | 2.49E-12 | 0.001139022 | 417.2982059 |
| 7 | rs17712208 | A | T | 365,950 | 0.0253079 | 0.151931 | finngen_R9_T2D | 0.0177717 | 1.93E-09 | 0.000805869 | 295.1438295 |
| 8 | rs348330 | A | G | 365,950 | 0.00711982 | -0.0490562 | finngen_R9_T2D | 0.644104 | 5.58E-12 | 0.001103308 | 404.1994419 |
| 9 | rs7563362 | G | A | 365,950 | 0.00972742 | 0.0657717 | finngen_R9_T2D | 0.854034 | 1.37E-11 | 0.001078537 | 395.1145576 |
| 10 | rs11124906 | C | G | 365,950 | 0.00706824 | -0.0393834 | finngen_R9_T2D | 0.386887 | 2.52E-08 | 0.000735836 | 269.4760267 |
| 11 | rs11899863 | T | C | 365,950 | 0.0162393 | -0.138821 | finngen_R9_T2D | 0.0488109 | 1.25E-17 | 0.001789468 | 656.0262893 |
| 12 | rs12713389 | A | T | 365,950 | 0.00682057 | -0.0386052 | finngen_R9_T2D | 0.515743 | 1.51E-08 | 0.000744442 | 272.6300128 |
| 13 | rs10084393 | A | C | 365,950 | 0.00820794 | -0.061283 | finngen_R9_T2D | 0.226837 | 8.25E-14 | 0.001317331 | 482.7106186 |
| 14 | rs35824707 | G | A | 365,950 | 0.0156771 | 0.105366 | finngen_R9_T2D | 0.0480585 | 1.81E-11 | 0.001015808 | 372.1107494 |
| 15 | rs13427924 | C | T | 365,950 | 0.00681282 | -0.0400364 | finngen_R9_T2D | 0.493163 | 4.19E-09 | 0.000801307 | 293.4717845 |
| 16 | rs74563586 | A | G | 365,950 | 0.0392902 | -0.232637 | finngen_R9_T2D | 0.00807881 | 3.20E-09 | 0.000867385 | 317.6935354 |
| 17 | rs13030400 | G | C | 365,950 | 0.00681734 | -0.0389073 | finngen_R9_T2D | 0.477254 | 1.15E-08 | 0.000755323 | 276.6177298 |
| 18 | rs17036160 | T | C | 365,950 | 0.00916178 | -0.0918512 | finngen_R9_T2D | 0.171956 | 1.18E-23 | 0.002402539 | 881.3216853 |
| 19 | rs35352848 | C | T | 365,950 | 0.00741446 | -0.0705068 | finngen_R9_T2D | 0.310129 | 1.92E-21 | 0.00212717 | 780.0931285 |
| 20 | rs3731503 | C | T | 365,950 | 0.00724711 | 0.0413463 | finngen_R9_T2D | 0.330055 | 1.16E-08 | 0.000756012 | 276.8704452 |
| 21 | rs9843558 | A | G | 365,950 | 0.0115121 | -0.0639821 | finngen_R9_T2D | 0.101294 | 2.73E-08 | 0.00074533 | 272.955299 |
| 22 | rs11720108 | T | C | 365,950 | 0.00902742 | -0.0761081 | finngen_R9_T2D | 0.176407 | 3.43E-17 | 0.00168314 | 616.9801464 |
| 23 | rs1604038 | T | C | 365,950 | 0.00774472 | -0.0538948 | finngen_R9_T2D | 0.263869 | 3.43E-12 | 0.001128411 | 413.406138 |
| 24 | rs13067541 | T | C | 365,950 | 0.0129962 | -0.0743936 | finngen_R9_T2D | 0.0789894 | 1.04E-08 | 0.000805257 | 294.9197298 |
| 25 | rs73061095 | G | T | 365,950 | 0.00732944 | 0.0956295 | finngen_R9_T2D | 0.307779 | 6.58E-39 | 0.003896705 | 1431.569779 |
| 26 | rs3887925 | T | C | 365,950 | 0.00684735 | 0.0483411 | finngen_R9_T2D | 0.463838 | 1.67E-12 | 0.001162319 | 425.8433494 |
| 27 | rs1046316 | G | A | 365,950 | 0.00703756 | 0.069643 | finngen_R9_T2D | 0.612848 | 4.34E-23 | 0.002301544 | 844.1882351 |
| 28 | rs12507026 | T | A | 365,950 | 0.00682924 | 0.0652671 | finngen_R9_T2D | 0.47262 | 1.21E-21 | 0.00212351 | 778.748041 |
| 29 | rs17008861 | T | C | 365,950 | 0.0139682 | -0.0827305 | finngen_R9_T2D | 0.0670829 | 3.17E-09 | 0.000856675 | 313.7673596 |
| 30 | rs62319060 | C | T | 365,950 | 0.00686469 | 0.0429167 | finngen_R9_T2D | 0.437531 | 4.06E-10 | 0.000906546 | 332.0498755 |
| 31 | rs7697171 | T | C | 365,950 | 0.00684438 | -0.0481932 | finngen_R9_T2D | 0.459958 | 1.91E-12 | 0.001153844 | 422.7348121 |
| 32 | rs6850761 | C | A | 365,950 | 0.00709212 | -0.0454342 | finngen_R9_T2D | 0.366615 | 1.49E-10 | 0.00095868 | 351.163766 |
| 33 | rs4862423 | T | C | 365,950 | 0.00701568 | 0.0452028 | finngen_R9_T2D | 0.382205 | 1.17E-10 | 0.000964942 | 353.459837 |
| 34 | rs78486128 | A | G | 365,950 | 0.0112443 | 0.0667625 | finngen_R9_T2D | 0.10351 | 2.89E-09 | 0.000827224 | 302.9714609 |
| 35 | rs6878122 | A | G | 365,950 | 0.00806625 | -0.0595912 | finngen_R9_T2D | 0.771826 | 1.49E-13 | 0.001250777 | 458.2924907 |
| 36 | rs35658696 | G | A | 365,950 | 0.014359 | 0.106064 | finngen_R9_T2D | 0.0577378 | 1.51E-13 | 0.001224047 | 448.4864232 |
| 37 | rs9505086 | C | T | 365,950 | 0.00687167 | 0.0626466 | finngen_R9_T2D | 0.444996 | 7.75E-20 | 0.001938551 | 710.7867516 |
| 38 | rs6931514 | G | A | 365,950 | 0.00719289 | 0.119851 | finngen_R9_T2D | 0.328486 | 2.46E-62 | 0.006337023 | 2333.810154 |
| 39 | rs9350408 | T | C | 365,950 | 0.00683082 | -0.0424767 | finngen_R9_T2D | 0.511342 | 5.02E-10 | 0.000901671 | 330.2624195 |
| 40 | rs1966 | T | C | 365,950 | 0.00813584 | 0.0483694 | finngen_R9_T2D | 0.224559 | 2.76E-09 | 0.000814799 | 298.4173068 |
| 41 | rs429150 | C | T | 365,950 | 0.00682391 | 0.0450307 | finngen_R9_T2D | 0.533624 | 4.14E-11 | 0.001009297 | 369.7233424 |
| 42 | rs4711750 | A | T | 365,950 | 0.00685804 | 0.042631 | finngen_R9_T2D | 0.498178 | 5.09E-10 | 0.000908689 | 332.8353712 |
| 43 | rs1665908 | T | A | 365,950 | 0.00696968 | -0.0428955 | finngen_R9_T2D | 0.415459 | 7.53E-10 | 0.00089371 | 327.3439352 |
| 44 | rs853974 | C | T | 365,950 | 0.00762886 | -0.0505198 | finngen_R9_T2D | 0.730996 | 3.54E-11 | 0.001003753 | 367.6905774 |
| 45 | rs12195206 | T | C | 365,950 | 0.00798883 | 0.0501421 | finngen_R9_T2D | 0.235762 | 3.46E-10 | 0.000906019 | 331.8566339 |
| 46 | rs618652 | G | T | 365,950 | 0.00682814 | -0.0418507 | finngen_R9_T2D | 0.481219 | 8.84E-10 | 0.000874505 | 320.3034479 |
| 47 | rs9371672 | G | C | 365,950 | 0.00721562 | 0.0457961 | finngen_R9_T2D | 0.334452 | 2.20E-10 | 0.000933685 | 341.9993079 |
| 48 | rs17168486 | T | C | 365,950 | 0.00851563 | 0.0496089 | finngen_R9_T2D | 0.197691 | 5.69E-09 | 0.000780688 | 285.9145861 |
| 49 | rs12113083 | C | G | 365,950 | 0.00692414 | 0.054507 | finngen_R9_T2D | 0.4125 | 3.49E-15 | 0.001440013 | 527.7297737 |
| 50 | rs498475 | A | G | 365,950 | 0.00711562 | -0.0702015 | finngen_R9_T2D | 0.645906 | 5.85E-23 | 0.002254295 | 826.8184838 |
| 51 | rs878521 | A | G | 365,950 | 0.00835164 | 0.0688112 | finngen_R9_T2D | 0.20609 | 1.73E-16 | 0.001549446 | 567.8965967 |
| 52 | rs34400892 | A | T | 365,950 | 0.0185638 | -0.110167 | finngen_R9_T2D | 0.0364534 | 2.95E-09 | 0.000852597 | 312.2724139 |
| 53 | rs3763432 | T | C | 365,950 | 0.00685305 | -0.0398479 | finngen_R9_T2D | 0.502934 | 6.08E-09 | 0.0007939 | 290.7570333 |
| 54 | rs35005436 | T | C | 365,950 | 0.0106304 | -0.0621371 | finngen_R9_T2D | 0.884426 | 5.06E-09 | 0.000789321 | 289.0785804 |
| 55 | rs79356898 | A | G | 365,950 | 0.014739 | 0.104377 | finngen_R9_T2D | 0.0554797 | 1.42E-12 | 0.001141787 | 418.3122179 |
| 56 | rs200641079 | C | T | 365,950 | 0.00885957 | -0.0811668 | finngen_R9_T2D | 0.18788 | 5.12E-20 | 0.002010424 | 737.1926124 |
| 57 | rs1646629 | T | C | 365,950 | 0.00686434 | 0.0517495 | finngen_R9_T2D | 0.435059 | 4.74E-14 | 0.001316417 | 482.3752656 |
| 58 | rs7788808 | C | T | 365,950 | 0.00795383 | 0.0576708 | finngen_R9_T2D | 0.753198 | 4.15E-13 | 0.001236516 | 453.0608159 |
| 59 | rs1182436 | C | T | 365,950 | 0.00974199 | 0.0738976 | finngen_R9_T2D | 0.853404 | 3.31E-14 | 0.001366367 | 500.7035233 |
| 60 | rs4240624 | A | G | 365,950 | 0.00968592 | -0.0608692 | finngen_R9_T2D | 0.857189 | 3.29E-10 | 0.000907117 | 332.2591469 |
| 61 | rs12549902 | A | G | 365,950 | 0.00691348 | 0.0589031 | finngen_R9_T2D | 0.583678 | 1.60E-17 | 0.0016862 | 618.1036212 |
| 62 | rs7845219 | C | T | 365,950 | 0.00680411 | -0.0408162 | finngen_R9_T2D | 0.508671 | 1.99E-09 | 0.000832731 | 304.9900635 |
| 63 | rs11558471 | G | A | 365,950 | 0.00704937 | -0.0818036 | finngen_R9_T2D | 0.377455 | 3.92E-31 | 0.003144928 | 1154.511108 |
| 64 | rs17772814 | A | G | 365,950 | 0.0116417 | -0.0804395 | finngen_R9_T2D | 0.103336 | 4.86E-12 | 0.001199085 | 439.3296833 |
| 65 | rs554186429 | C | G | 365,950 | 0.0151277 | -0.0956941 | finngen_R9_T2D | 0.0590239 | 2.52E-10 | 0.001017201 | 372.6217342 |
| 66 | rs10974438 | C | A | 365,950 | 0.00704162 | 0.0431823 | finngen_R9_T2D | 0.374208 | 8.65E-10 | 0.000873343 | 319.8773153 |
| 67 | rs10965247 | G | A | 365,950 | 0.00964083 | -0.107939 | finngen_R9_T2D | 0.150767 | 4.27E-29 | 0.002983458 | 1095.057554 |
| 68 | rs7018475 | G | T | 365,950 | 0.00753617 | 0.0989339 | finngen_R9_T2D | 0.27897 | 2.28E-39 | 0.003937595 | 1446.651499 |
| 69 | rs12001437 | C | T | 365,950 | 0.00712404 | 0.041023 | finngen_R9_T2D | 0.352183 | 8.49E-09 | 0.000767902 | 281.2280013 |
| 70 | rs17791633 | C | T | 365,950 | 0.0103666 | -0.0618274 | finngen_R9_T2D | 0.126933 | 2.46E-09 | 0.000847255 | 310.3141453 |
| 71 | rs9410573 | C | T | 365,950 | 0.00692417 | -0.0410166 | finngen_R9_T2D | 0.416847 | 3.15E-09 | 0.000817916 | 299.5596018 |
| 72 | rs607409 | T | A | 365,950 | 0.00701026 | 0.0488966 | finngen_R9_T2D | 0.608971 | 3.06E-12 | 0.001138657 | 417.1642372 |
| 73 | rs960312 | A | G | 365,950 | 0.00788619 | 0.0439186 | finngen_R9_T2D | 0.745798 | 2.56E-08 | 0.000731353 | 267.8331106 |
| 74 | rs8176645 | T | A | 365,950 | 0.00683066 | -0.0567465 | finngen_R9_T2D | 0.541902 | 9.76E-17 | 0.001598775 | 586.0053469 |
| 75 | rs28624681 | T | C | 365,950 | 0.00755133 | -0.076247 | finngen_R9_T2D | 0.293087 | 5.69E-24 | 0.002409007 | 883.7000905 |
| 76 | rs35529305 | A | G | 365,950 | 0.0131158 | -0.0734098 | finngen_R9_T2D | 0.0769342 | 2.18E-08 | 0.000765403 | 280.3122617 |
| 77 | rs11257659 | T | C | 365,950 | 0.00769491 | 0.0811613 | finngen_R9_T2D | 0.265372 | 5.22E-26 | 0.002568328 | 942.2947763 |
| 78 | rs190611672 | A | G | 365,950 | 0.0247333 | 0.140569 | finngen_R9_T2D | 0.0187094 | 1.32E-08 | 0.000725549 | 265.7059007 |
| 79 | rs72807614 | A | G | 365,950 | 0.00974009 | 0.0721667 | finngen_R9_T2D | 0.141653 | 1.27E-13 | 0.001266463 | 464.0471431 |
| 80 | rs2394576 | C | T | 365,950 | 0.00686602 | 0.0424454 | finngen_R9_T2D | 0.511141 | 6.33E-10 | 0.000900359 | 329.781406 |
| 81 | rs703980 | A | G | 365,950 | 0.00680829 | -0.048787 | finngen_R9_T2D | 0.478957 | 7.73E-13 | 0.001187978 | 435.2551615 |
| 82 | rs10882099 | C | T | 365,950 | 0.00682772 | -0.0691246 | finngen_R9_T2D | 0.476862 | 4.32E-24 | 0.002383989 | 874.5007958 |
| 83 | rs10885123 | T | C | 365,950 | 0.0101393 | -0.0591593 | finngen_R9_T2D | 0.133879 | 5.39E-09 | 0.000811647 | 297.2617924 |
| 84 | rs114322470 | G | T | 365,950 | 0.02366 | -0.224936 | finngen_R9_T2D | 0.0233266 | 1.96E-21 | 0.002305413 | 845.61075 |
| 85 | rs34872471 | C | T | 365,950 | 0.00830097 | 0.254237 | finngen_R9_T2D | 0.202205 | 1.00E-200 | 0.020854064 | 7794.040514 |
| 86 | rs61872821 | G | A | 365,950 | 0.0185694 | 0.11168 | finngen_R9_T2D | 0.0358766 | 1.81E-09 | 0.000862829 | 316.0232026 |
| 87 | rs7099964 | C | G | 365,950 | 0.0132258 | 0.0812819 | finngen_R9_T2D | 0.075331 | 7.96E-10 | 0.000920402 | 337.129695 |
| 88 | rs78721871 | A | G | 365,950 | 0.0180437 | 0.113577 | finngen_R9_T2D | 0.0359248 | 3.08E-10 | 0.000893544 | 327.2831639 |
| 89 | rs10770143 | T | C | 365,950 | 0.00716832 | -0.0666259 | finngen_R9_T2D | 0.660446 | 1.48E-20 | 0.001990959 | 730.0409817 |
| 90 | rs2521247 | T | C | 365,950 | 0.00703788 | 0.0459196 | finngen_R9_T2D | 0.609199 | 6.82E-11 | 0.001004017 | 367.7872524 |
| 91 | rs74046911 | T | C | 365,950 | 0.0134382 | -0.206625 | finngen_R9_T2D | 0.07601 | 2.38E-53 | 0.005996996 | 2207.828897 |
| 92 | rs17596617 | T | C | 365,950 | 0.00731609 | 0.0402577 | finngen_R9_T2D | 0.311757 | 3.74E-08 | 0.000695482 | 254.6873987 |
| 93 | rs7935034 | C | T | 365,950 | 0.00745253 | -0.0412498 | finngen_R9_T2D | 0.306487 | 3.11E-08 | 0.000723336 | 264.8951381 |
| 94 | rs12420590 | G | C | 365,950 | 0.00793739 | 0.0464028 | finngen_R9_T2D | 0.2476 | 5.03E-09 | 0.000802265 | 293.8229632 |
| 95 | rs72932523 | A | G | 365,950 | 0.00925186 | -0.0518791 | finngen_R9_T2D | 0.16795 | 2.05E-08 | 0.000752219 | 275.4802579 |
| 96 | rs7109575 | A | G | 365,950 | 0.00810131 | -0.0903571 | finngen_R9_T2D | 0.237142 | 6.89E-29 | 0.002953975 | 1084.203782 |
| 97 | rs10830963 | G | C | 365,950 | 0.00709002 | 0.107579 | finngen_R9_T2D | 0.357166 | 5.31E-52 | 0.005314396 | 1955.183377 |
| 98 | rs897558 | G | A | 365,950 | 0.00682545 | 0.038199 | finngen_R9_T2D | 0.47749 | 2.19E-08 | 0.000728103 | 266.6420102 |
| 99 | rs7948351 | T | C | 365,950 | 0.00960472 | -0.0550677 | finngen_R9_T2D | 0.151962 | 9.84E-09 | 0.000781581 | 286.2418514 |
| 100 | rs4937325 | C | T | 365,950 | 0.007223 | -0.0478799 | finngen_R9_T2D | 0.667232 | 3.38E-11 | 0.001018017 | 372.9208027 |
| 101 | rs112108223 | A | G | 365,950 | 0.0249156 | -0.354533 | finngen_R9_T2D | 0.0225891 | 6.02E-46 | 0.005550338 | 2042.471505 |
| 102 | rs73040004 | C | T | 365,950 | 0.00819527 | 0.0703653 | finngen_R9_T2D | 0.221558 | 9.00E-18 | 0.001707893 | 626.0694606 |
| 103 | rs76895963 | G | T | 365,950 | 0.0224675 | -0.508464 | finngen_R9_T2D | 0.0306203 | 2.14E-113 | 0.01534807 | 5704.143186 |
| 104 | rs78470967 | A | T | 365,950 | 0.0182222 | -0.1964 | finngen_R9_T2D | 0.0397517 | 4.37E-27 | 0.002944776 | 1080.817531 |
| 105 | rs61909599 | C | G | 365,950 | 0.0113239 | 0.0686879 | finngen_R9_T2D | 0.099409 | 1.31E-09 | 0.00084478 | 309.4070445 |
| 106 | rs10466811 | A | G | 365,950 | 0.00808935 | -0.0512322 | finngen_R9_T2D | 0.23707 | 2.40E-10 | 0.000949461 | 347.7836963 |
| 107 | rs11052566 | T | C | 365,950 | 0.0126018 | 0.0758584 | finngen_R9_T2D | 0.0774955 | 1.75E-09 | 0.000822777 | 301.3416647 |
| 108 | rs11169182 | T | C | 365,950 | 0.00683204 | 0.0417543 | finngen_R9_T2D | 0.493559 | 9.87E-10 | 0.000871566 | 319.2261078 |
| 109 | rs73115426 | C | T | 365,950 | 0.0223095 | 0.199451 | finngen_R9_T2D | 0.0222602 | 3.88E-19 | 0.001731629 | 634.7852904 |
| 110 | rs1397566 | G | A | 365,950 | 0.0068898 | -0.0416204 | finngen_R9_T2D | 0.428008 | 1.53E-09 | 0.000848173 | 310.650622 |
| 111 | rs1988138 | T | C | 365,950 | 0.00800576 | -0.046698 | finngen_R9_T2D | 0.765392 | 5.44E-09 | 0.000783165 | 286.8223096 |
| 112 | rs74628648 | T | C | 365,950 | 0.01288 | -0.0784778 | finngen_R9_T2D | 0.0780339 | 1.11E-09 | 0.00088618 | 324.5834053 |
| 113 | rs56348580 | C | G | 365,950 | 0.00761837 | -0.0801679 | finngen_R9_T2D | 0.284494 | 6.77E-26 | 0.00261648 | 960.0074247 |
| 114 | rs73224247 | C | T | 365,950 | 0.0115306 | -0.0735272 | finngen_R9_T2D | 0.101988 | 1.81E-10 | 0.000990278 | 362.74959 |
| 115 | rs141433349 | A | G | 365,950 | 0.0195216 | 0.110395 | finngen_R9_T2D | 0.0312835 | 1.56E-08 | 0.000738654 | 270.5086307 |
| 116 | rs488321 | C | T | 365,950 | 0.0115036 | -0.0861415 | finngen_R9_T2D | 0.90532 | 6.98E-14 | 0.001272082 | 466.1089129 |
| 117 | rs963740 | T | A | 365,950 | 0.00838674 | -0.0661672 | finngen_R9_T2D | 0.212166 | 3.03E-15 | 0.001463612 | 536.3910218 |
| 118 | rs57898296 | G | C | 365,950 | 0.00708119 | 0.0534958 | finngen_R9_T2D | 0.360922 | 4.20E-14 | 0.00132019 | 483.7597149 |
| 119 | rs7995767 | C | T | 365,950 | 0.0192221 | -0.10831 | finngen_R9_T2D | 0.968265 | 1.75E-08 | 0.000720941 | 264.0173303 |
| 120 | rs4981709 | G | A | 365,950 | 0.00693674 | 0.0390506 | finngen_R9_T2D | 0.402363 | 1.81E-08 | 0.0007334 | 268.5832587 |
| 121 | rs35120418 | A | C | 365,950 | 0.00687763 | 0.040337 | finngen_R9_T2D | 0.44307 | 4.49E-09 | 0.00080299 | 294.0887395 |
| 122 | rs73347525 | G | A | 365,950 | 0.00832412 | -0.0523298 | finngen_R9_T2D | 0.220948 | 3.25E-10 | 0.000942724 | 345.31358 |
| 123 | rs28798715 | A | C | 365,950 | 0.0107471 | -0.0647065 | finngen_R9_T2D | 0.116637 | 1.74E-09 | 0.000862783 | 316.0061982 |
| 124 | rs11631200 | A | G | 365,950 | 0.00684032 | 0.0423933 | finngen_R9_T2D | 0.493005 | 5.73E-10 | 0.00089842 | 329.0706712 |
| 125 | rs2682907 | A | G | 365,950 | 0.00694845 | -0.0602319 | finngen_R9_T2D | 0.412874 | 4.38E-18 | 0.001758863 | 644.7864236 |
| 126 | rs2034084 | G | C | 365,950 | 0.00719139 | 0.0494996 | finngen_R9_T2D | 0.337147 | 5.85E-12 | 0.001095141 | 401.203906 |
| 127 | rs4374177 | G | A | 365,950 | 0.0107479 | -0.0690122 | finngen_R9_T2D | 0.116458 | 1.35E-10 | 0.000980118 | 359.0240263 |
| 128 | rs66502159 | T | C | 365,950 | 0.0117369 | -0.112855 | finngen_R9_T2D | 0.0963757 | 6.88E-22 | 0.002218335 | 813.5999463 |
| 129 | rs3096301 | T | C | 365,950 | 0.00712506 | 0.0399613 | finngen_R9_T2D | 0.6249 | 2.04E-08 | 0.000748629 | 274.1646301 |
| 130 | rs8071043 | C | T | 365,950 | 0.00734389 | 0.0702501 | finngen_R9_T2D | 0.30881 | 1.11E-21 | 0.002106748 | 772.5880443 |
| 131 | rs739753 | T | A | 365,950 | 0.00809855 | -0.0625112 | finngen_R9_T2D | 0.24097 | 1.17E-14 | 0.001429445 | 523.8515206 |
| 132 | rs35895680 | A | C | 365,950 | 0.00742105 | -0.0419803 | finngen_R9_T2D | 0.310306 | 1.54E-08 | 0.000754341 | 276.2579597 |
| 133 | rs4968896 | G | T | 365,950 | 0.00683265 | 0.0431248 | finngen_R9_T2D | 0.491659 | 2.76E-10 | 0.000929615 | 340.5074424 |
| 134 | rs7226371 | G | A | 365,950 | 0.00893201 | 0.0574686 | finngen_R9_T2D | 0.173083 | 1.24E-10 | 0.000945383 | 346.2882148 |
| 135 | rs56192168 | G | A | 365,950 | 0.00928492 | -0.0568257 | finngen_R9_T2D | 0.840977 | 9.34E-10 | 0.000863701 | 316.3430413 |
| 136 | rs62092443 | T | C | 365,950 | 0.0117407 | 0.0698079 | finngen_R9_T2D | 0.0915721 | 2.75E-09 | 0.000810761 | 296.9370641 |
| 137 | rs2894553 | T | C | 365,950 | 0.0123906 | -0.0759144 | finngen_R9_T2D | 0.085166 | 8.97E-10 | 0.000898022 | 328.9246563 |
| 138 | rs2303700 | C | T | 365,950 | 0.00728101 | -0.0480432 | finngen_R9_T2D | 0.67481 | 4.16E-11 | 0.001013007 | 371.0838768 |
| 139 | rs10404726 | T | C | 365,950 | 0.00681874 | -0.0422001 | finngen_R9_T2D | 0.523887 | 6.06E-10 | 0.000888392 | 325.3943371 |
| 140 | rs8100204 | A | G | 365,950 | 0.00932867 | 0.0748815 | finngen_R9_T2D | 0.158441 | 9.99E-16 | 0.00149531 | 548.0251367 |
| 141 | rs10408179 | C | T | 365,950 | 0.00685658 | -0.0567973 | finngen_R9_T2D | 0.447143 | 1.19E-16 | 0.001594941 | 584.597874 |
| 142 | rs6142129 | G | A | 365,950 | 0.00730934 | -0.0474735 | finngen_R9_T2D | 0.328749 | 8.31E-11 | 0.000994677 | 364.3623212 |
| 143 | rs6030812 | A | G | 365,950 | 0.00942153 | -0.0514736 | finngen_R9_T2D | 0.846419 | 4.67E-08 | 0.000688846 | 252.255486 |
| 144 | rs112650492 | G | C | 365,950 | 0.0180696 | 0.158855 | finngen_R9_T2D | 0.0352261 | 1.48E-18 | 0.001715228 | 628.7627645 |
| 145 | rs77735929 | A | T | 365,950 | 0.0164803 | 0.147664 | finngen_R9_T2D | 0.0433555 | 3.25E-19 | 0.001808731 | 663.100938 |
| 146 | rs45551238 | T | C | 365,950 | 0.0164557 | -0.173379 | finngen_R9_T2D | 0.0490352 | 5.89E-26 | 0.002803467 | 1028.807197 |
| 147 | rs8126001 | T | C | 365,950 | 0.00687928 | -0.0377239 | finngen_R9_T2D | 0.521916 | 4.17E-08 | 0.000710179 | 260.0733791 |
| 148 | rs142682088 | A | G | 365,950 | 0.017457 | -0.110789 | finngen_R9_T2D | 0.0412275 | 2.20E-10 | 0.000970344 | 355.4404478 |

SNP, single nucleotide polymorphism; EA, effect allele; OA, other allele; EAF, effect allele frequency; SE, standard error; T2D, type 2 diabetes mellitus.

**Supplementary Table S1.4** Characteristics of instrumental variables for GDM.

|  | **SNP** | **EA** | **OA** | **Samplesize** | **SE** | **β** | **id.exposure** | **EAF** | ***p* value** | **R^2^** | **F - statistic** |
| --- | --- | --- | --- | --- | --- | --- | --- | --- | --- | --- | --- |
| 1 | rs12125375 | C | T | 190,879 | 0.0150437 | -0.0722744 | finngen_R8_GEST_DIABETES | 0.334299 | 1.55E-06 | 0.002324948 | 444.8132964 |
| 2 | rs77315096 | G | T | 190,879 | 0.0552765 | -0.255231 | finngen_R8_GEST_DIABETES | 0.0192551 | 3.89E-06 | 0.00246036 | 470.7844554 |
| 3 | rs1402837 | T | C | 190,879 | 0.0184198 | 0.088837 | finngen_R8_GEST_DIABETES | 0.169918 | 1.41E-06 | 0.002226272 | 425.8921977 |
| 4 | rs112512273 | T | C | 190,879 | 0.0319754 | 0.147122 | finngen_R8_GEST_DIABETES | 0.0483195 | 4.20E-06 | 0.001990668 | 380.7306468 |
| 5 | rs11732913 | C | G | 190,879 | 0.0276605 | -0.128325 | finngen_R8_GEST_DIABETES | 0.0743199 | 3.50E-06 | 0.002265784 | 433.4682779 |
| 6 | rs7722200 | C | T | 190,879 | 0.0152551 | -0.144082 | finngen_R8_GEST_DIABETES | 0.31561 | 3.56E-21 | 0.008968171 | 1727.308301 |
| 7 | rs58667885 | A | G | 190,879 | 0.0532941 | 0.249375 | finngen_R8_GEST_DIABETES | 0.015893 | 2.88E-06 | 0.001945289 | 372.0345533 |
| 8 | rs144093119 | A | T | 190,879 | 0.0269934 | -0.123562 | finngen_R8_GEST_DIABETES | 0.0812873 | 4.71E-06 | 0.002280354 | 436.2619631 |
| 9 | rs7756992 | G | A | 190,879 | 0.014676 | 0.102687 | finngen_R8_GEST_DIABETES | 0.332859 | 2.62E-12 | 0.004683159 | 898.1132663 |
| 10 | rs2256965 | G | A | 190,879 | 0.0141651 | 0.0766284 | finngen_R8_GEST_DIABETES | 0.56731 | 6.31E-08 | 0.002882749 | 551.8412726 |
| 11 | rs9275373 | A | G | 190,879 | 0.0208377 | 0.158059 | finngen_R8_GEST_DIABETES | 0.120313 | 3.32E-14 | 0.005288216 | 1014.765102 |
| 12 | rs75394590 | G | T | 190,879 | 0.0467293 | -0.216968 | finngen_R8_GEST_DIABETES | 0.0254331 | 3.43E-06 | 0.002333632 | 446.478542 |
| 13 | rs12663869 | T | C | 190,879 | 0.0400559 | 0.185041 | finngen_R8_GEST_DIABETES | 0.0289602 | 3.85E-06 | 0.00192577 | 368.2945355 |
| 14 | rs12199962 | T | A | 190,879 | 0.123815 | -0.594543 | finngen_R8_GEST_DIABETES | 0.00461263 | 1.57E-06 | 0.003245916 | 621.5883384 |
| 15 | rs9390405 | G | C | 190,879 | 0.0150457 | -0.0734196 | finngen_R8_GEST_DIABETES | 0.689877 | 1.06E-06 | 0.002306533 | 441.2819246 |
| 16 | rs143769538 | G | C | 190,879 | 0.0380108 | -0.175903 | finngen_R8_GEST_DIABETES | 0.0390667 | 3.70E-06 | 0.002323146 | 444.4676565 |
| 17 | rs10758593 | A | G | 190,879 | 0.01408 | 0.0674627 | finngen_R8_GEST_DIABETES | 0.438671 | 1.66E-06 | 0.002241371 | 428.7873315 |
| 18 | rs1333051 | T | A | 190,879 | 0.0227193 | -0.132373 | finngen_R8_GEST_DIABETES | 0.115008 | 5.66E-09 | 0.003566943 | 683.2846877 |
| 19 | rs28624681 | T | C | 190,879 | 0.0156154 | -0.0780739 | finngen_R8_GEST_DIABETES | 0.292718 | 5.74E-07 | 0.002523968 | 482.986409 |
| 20 | rs2237897 | T | C | 190,879 | 0.026516 | -0.133555 | finngen_R8_GEST_DIABETES | 0.0816536 | 4.73E-07 | 0.002675052 | 511.975385 |
| 21 | rs7933420 | A | T | 190,879 | 0.0141464 | -0.0714026 | finngen_R8_GEST_DIABETES | 0.454042 | 4.48E-07 | 0.002527629 | 483.6888049 |
| 22 | rs1346 | T | A | 190,879 | 0.0217018 | 0.10034 | finngen_R8_GEST_DIABETES | 0.113587 | 3.77E-06 | 0.002027416 | 387.7733202 |
| 23 | rs613937 | G | A | 190,879 | 0.0161994 | -0.0780174 | finngen_R8_GEST_DIABETES | 0.258767 | 1.46E-06 | 0.002334945 | 446.7303086 |
| 24 | rs141865255 | G | T | 190,879 | 0.0330617 | 0.155666 | finngen_R8_GEST_DIABETES | 0.04329 | 2.50E-06 | 0.002007176 | 383.8942513 |
| 25 | rs76349839 | C | A | 190,879 | 0.040959 | -0.205991 | finngen_R8_GEST_DIABETES | 0.034055 | 4.93E-07 | 0.002791642 | 534.3520278 |
| 26 | rs7123869 | C | T | 190,879 | 0.0458942 | 0.295913 | finngen_R8_GEST_DIABETES | 0.0200881 | 1.14E-10 | 0.003447339 | 660.2939627 |
| 27 | rs10830963 | G | C | 190,879 | 0.014106 | 0.36359 | finngen_R8_GEST_DIABETES | 0.357465 | 1.67E-146 | 0.060727328 | 12340.87873 |
| 28 | rs74829505 | A | G | 190,879 | 0.0434287 | -0.217832 | finngen_R8_GEST_DIABETES | 0.0298091 | 5.28E-07 | 0.002744602 | 525.3232461 |
| 29 | rs755110 | T | C | 190,879 | 0.129841 | -0.606763 | finngen_R8_GEST_DIABETES | 0.00450479 | 2.97E-06 | 0.003302037 | 632.3709781 |
| 30 | rs76895963 | G | T | 190,879 | 0.044236 | -0.224212 | finngen_R8_GEST_DIABETES | 0.0306892 | 4.01E-07 | 0.002990862 | 572.5992591 |
| 31 | rs6538804 | G | C | 190,879 | 0.014778 | -0.0794568 | finngen_R8_GEST_DIABETES | 0.355976 | 7.59E-08 | 0.002894776 | 554.150228 |
| 32 | rs6562107 | C | T | 190,879 | 0.0189264 | -0.0880244 | finngen_R8_GEST_DIABETES | 0.173673 | 3.31E-06 | 0.002223926 | 425.4425347 |
| 33 | rs186865268 | T | C | 190,879 | 0.177636 | -0.82615 | finngen_R8_GEST_DIABETES | 0.00251539 | 3.31E-06 | 0.00342499 | 655.9986595 |
| 34 | rs7227997 | A | G | 190,879 | 0.0161689 | -0.0741786 | finngen_R8_GEST_DIABETES | 0.750124 | 4.48E-06 | 0.002062742 | 394.5438061 |
| 35 | rs1055080 | A | G | 190,879 | 0.042291 | -0.20224 | finngen_R8_GEST_DIABETES | 0.0318209 | 1.73E-06 | 0.002520184 | 482.26057 |
| 36 | rs6021276 | C | T | 190,879 | 0.0143128 | 0.0678764 | finngen_R8_GEST_DIABETES | 0.583403 | 2.11E-06 | 0.002239507 | 428.4298179 |
| 37 | rs2187240 | A | G | 190,879 | 0.0212562 | 0.0988775 | finngen_R8_GEST_DIABETES | 0.118131 | 3.29E-06 | 0.002037009 | 389.6117728 |
| 38 | rs13053342 | A | G | 190,879 | 0.0141629 | 0.0671075 | finngen_R8_GEST_DIABETES | 0.411615 | 2.16E-06 | 0.002181348 | 417.2793409 |

SNP, single nucleotide polymorphism; EA, effect allele; OA, other allele; EAF, effect allele frequency; SE, standard error; GDM, gestational diabetes mellitus.

**Supplementary Table S2.1** Information of identified SNPs in exposure (ASD) and outcomes (T1DM).

|  |  | | | **Exposure (ASD)** | | |  | **Outcome (T1DM)** | | | | |
| --- | --- | --- | --- | --- | --- | --- | --- | --- | --- | --- | --- | --- |
|  | **SNP** | **EA** | **OA** | **β** | **SE** | ***p* value** |  | **Case** | **Control** | **β** | **SE** | ***p* value** |
| 1 | rs10110094 | G | A | -0.0906996 | 0.0191 | 2.05E-06 |  | 3,049 | 279,760 | 0.0171135 | 0.0401999 | 0.670318 |
| 2 | rs11185408 | A | G | -0.0686965 | 0.0138 | 6.42E-07 |  | 3,049 | 279,760 | 0.00404491 | 0.0265434 | 0.87888 |
| 3 | rs144911765 | C | T | 0.190096 | 0.0403 | 2.39E-06 |  | 3,049 | 279,760 | 0.0683007 | 0.060925 | 0.262261 |
| 4 | rs149923766 | G | T | 0.237306 | 0.0484 | 9.44E-07 |  | 3,049 | 279,760 | 0.0959583 | 0.0884104 | 0.277756 |
| 5 | rs16879023 | A | G | -0.0957953 | 0.0201 | 1.88E-06 |  | 3,049 | 279,760 | -0.039247 | 0.0411325 | 0.340003 |
| 6 | rs2224274 | T | C | 0.0709989 | 0.0138 | 2.68E-07 |  | 3,049 | 279,760 | -0.00870943 | 0.0268953 | 0.746069 |
| 7 | rs2391769 | G | A | 0.0769026 | 0.0145 | 1.14E-07 |  | 3,049 | 279,760 | -0.00755134 | 0.0290097 | 0.794629 |
| 8 | rs2635182 | T | C | 0.0669014 | 0.014 | 1.76E-06 |  | 3,049 | 279,760 | 0.0541275 | 0.0271565 | 0.0462434 |
| 9 | rs28729902 | G | A | 0.0839035 | 0.0178 | 2.43E-06 |  | 3,049 | 279,760 | -0.0261278 | 0.031905 | 0.41283 |
| 10 | rs292441 | A | G | -0.0724954 | 0.0149 | 1.14E-06 |  | 3,049 | 279,760 | -0.0271144 | 0.0297625 | 0.362281 |
| 11 | rs35404050 | T | C | 0.0843044 | 0.0176 | 1.67E-06 |  | 3,049 | 279,760 | 0.0261602 | 0.0345835 | 0.449388 |
| 12 | rs45595836 | T | C | 0.138996 | 0.0272 | 3.22E-07 |  | 3,049 | 279,760 | -0.0354731 | 0.0606988 | 0.558944 |
| 13 | rs4750990 | C | T | 0.0680968 | 0.0141 | 1.37E-06 |  | 3,049 | 279,760 | -0.00814451 | 0.0268438 | 0.761583 |
| 14 | rs644552 | A | G | 0.159403 | 0.0346 | 4.08E-06 |  | 3,049 | 279,760 | 0.0745047 | 0.0659076 | 0.25829 |
| 15 | rs6692705 | G | A | -0.0656005 | 0.0141 | 3.28E-06 |  | 3,049 | 279,760 | -0.0330494 | 0.0275116 | 0.22964 |
| 16 | rs76397219 | G | A | 0.140297 | 0.0303 | 3.65E-06 |  | 3,049 | 279,760 | -0.0272927 | 0.0640499 | 0.670023 |
| 17 | rs77691144 | C | T | 0.207406 | 0.0435 | 1.86E-06 |  | 3,049 | 279,760 | 0.00493093 | 0.122992 | 0.96802 |
| 18 | rs7783557 | C | T | -0.0670042 | 0.0146 | 4.45E-06 |  | 3,049 | 279,760 | -0.0315665 | 0.0282011 | 0.262997 |
| 19 | rs78058104 | A | G | 0.187898 | 0.0397 | 2.21E-06 |  | 3,049 | 279,760 | 0.027629 | 0.0809549 | 0.732887 |
| 20 | rs78653484 | T | C | -0.176296 | 0.0385 | 4.67E-06 |  | 3,049 | 279,760 | 0.0217137 | 0.0565439 | 0.700968 |
| 21 | rs78827416 | A | G | 0.130502 | 0.0266 | 9.29E-07 |  | 3,049 | 279,760 | -0.0347206 | 0.0429776 | 0.419162 |
| 22 | rs79940520 | G | A | 0.0953992 | 0.0207 | 4.05E-06 |  | 3,049 | 279,760 | -0.0158787 | 0.0420107 | 0.705454 |
| 23 | rs9366877 | G | A | -0.0684994 | 0.0139 | 8.31E-07 |  | 3,049 | 279,760 | -0.00339726 | 0.0272216 | 0.900682 |
| 24 | rs9389208 | T | C | 0.0672006 | 0.0144 | 3.06E-06 |  | 3,049 | 279,760 | -0.00421972 | 0.0265498 | 0.873719 |

SNP, single nucleotide polymorphism; EA, effect allele; OA, other allele; SE, standard error; ASD, autism spectrum disorder; T1DM, type 1 diabetes mellitus.

**Supplementary Table S2.2** Information of identified SNPs in exposure (ASD) and outcomes (T2DM).

|  |  | | | **Exposure (ASD)** | | |  | **Outcome (T2DM)** | | | | |
| --- | --- | --- | --- | --- | --- | --- | --- | --- | --- | --- | --- | --- |
|  | **SNP** | **EA** | **OA** | **β** | **SE** | ***p* value** |  | **Case** | **Control** | **β** | **SE** | ***p* value** |
| 1 | rs10110094 | G | A | -0.0906996 | 0.0191 | 2.05E-06 |  | 57,698 | 308,252 | -0.00808136 | 0.010335 | 0.434248 |
| 2 | rs11185408 | A | G | -0.0686965 | 0.0138 | 6.42E-07 |  | 57,698 | 308,252 | 0.00508542 | 0.00681451 | 0.455508 |
| 3 | rs144911765 | C | T | 0.190096 | 0.0403 | 2.39E-06 |  | 57,698 | 308,252 | -0.00304912 | 0.01547 | 0.84375 |
| 4 | rs149923766 | G | T | 0.237306 | 0.0484 | 9.44E-07 |  | 57,698 | 308,252 | 0.0136017 | 0.0227719 | 0.550304 |
| 5 | rs16879023 | A | G | -0.0957953 | 0.0201 | 1.88E-06 |  | 57,698 | 308,252 | -0.00942292 | 0.0106207 | 0.374962 |
| 6 | rs2224274 | T | C | 0.0709989 | 0.0138 | 2.68E-07 |  | 57,698 | 308,252 | 0.00536302 | 0.00685846 | 0.43424 |
| 7 | rs2391769 | G | A | 0.0769026 | 0.0145 | 1.14E-07 |  | 57,698 | 308,252 | 0.00109149 | 0.00743469 | 0.883282 |
| 8 | rs2635182 | T | C | 0.0669014 | 0.014 | 1.76E-06 |  | 57,698 | 308,252 | 0.0140647 | 0.00696673 | 0.0435051 |
| 9 | rs28729902 | G | A | 0.0839035 | 0.0178 | 2.43E-06 |  | 57,698 | 308,252 | -0.00652013 | 0.00822113 | 0.427723 |
| 10 | rs35404050 | T | C | 0.0843044 | 0.0176 | 1.67E-06 |  | 57,698 | 308,252 | 0.0161414 | 0.00883607 | 0.0677345 |
| 11 | rs45595836 | T | C | 0.138996 | 0.0272 | 3.22E-07 |  | 57,698 | 308,252 | 0.0210293 | 0.0155369 | 0.175894 |
| 12 | rs4750990 | C | T | 0.0680968 | 0.0141 | 1.37E-06 |  | 57,698 | 308,252 | 0.00711491 | 0.00688286 | 0.301269 |
| 13 | rs644552 | A | G | 0.159403 | 0.0346 | 4.08E-06 |  | 57,698 | 308,252 | 0.0133439 | 0.0166237 | 0.422146 |
| 14 | rs76397219 | G | A | 0.140297 | 0.0303 | 3.65E-06 |  | 57,698 | 308,252 | -0.00842381 | 0.0164313 | 0.608183 |
| 15 | rs77691144 | C | T | 0.207406 | 0.0435 | 1.86E-06 |  | 57,698 | 308,252 | 0.0645487 | 0.0312366 | 0.0387864 |
| 16 | rs78058104 | A | G | 0.187898 | 0.0397 | 2.21E-06 |  | 57,698 | 308,252 | -0.00120236 | 0.0203483 | 0.952882 |
| 17 | rs78827416 | A | G | 0.130502 | 0.0266 | 9.29E-07 |  | 57,698 | 308,252 | -0.00491508 | 0.0111317 | 0.658822 |
| 18 | rs79940520 | G | A | 0.0953992 | 0.0207 | 4.05E-06 |  | 57,698 | 308,252 | 0.00478891 | 0.0106855 | 0.654032 |
| 19 | rs9366877 | G | A | -0.0684994 | 0.0139 | 8.31E-07 |  | 57,698 | 308,252 | 0.00236853 | 0.0070752 | 0.737802 |
| 20 | rs9389208 | T | C | 0.0672006 | 0.0144 | 3.06E-06 |  | 57,698 | 308,252 | 0.0151575 | 0.00689534 | 0.0279332 |

SNP, single nucleotide polymorphism; EA, effect allele; OA, other allele; SE, standard error; ASD, autism spectrum disorder; T2DM, type 2 diabetes mellitus.

**Supplementary Table S2.3** Information of identified SNPs in exposure (ASD) and outcomes (GDM).

|  |  | | | **Exposure (ASD)** | | |  | **Outcome (GDM)** | | | | |
| --- | --- | --- | --- | --- | --- | --- | --- | --- | --- | --- | --- | --- |
|  | **SNP** | **EA** | **OA** | **β** | **SE** | ***p* value** |  | **Case** | **Control** | **β** | **SE** | ***p* value** |
| 1 | rs10110094 | G | A | -0.0906996 | 0.0191 | 2.05E-06 |  | 11,279 | 179,600 | 0.00987916 | 0.0212936 | 0.642683 |
| 2 | rs11185408 | A | G | -0.0686965 | 0.0138 | 6.42E-07 |  | 11,279 | 179,600 | -0.00838946 | 0.0140642 | 0.550833 |
| 3 | rs144911765 | C | T | 0.190096 | 0.0403 | 2.39E-06 |  | 11,279 | 179,600 | 0.00894214 | 0.0319064 | 0.779277 |
| 4 | rs149923766 | G | T | 0.237306 | 0.0484 | 9.44E-07 |  | 11,279 | 179,600 | -0.0118854 | 0.0466529 | 0.798907 |
| 5 | rs16879023 | A | G | -0.0957953 | 0.0201 | 1.88E-06 |  | 11,279 | 179,600 | 0.038252 | 0.0219971 | 0.0820427 |
| 6 | rs2224274 | T | C | 0.0709989 | 0.0138 | 2.68E-07 |  | 11,279 | 179,600 | -0.00459759 | 0.014138 | 0.745034 |
| 7 | rs2391769 | G | A | 0.0769026 | 0.0145 | 1.14E-07 |  | 11,279 | 179,600 | -0.00483027 | 0.0153498 | 0.753005 |
| 8 | rs2635182 | T | C | 0.0669014 | 0.014 | 1.76E-06 |  | 11,279 | 179,600 | 0.0281067 | 0.0143272 | 0.0497898 |
| 9 | rs28729902 | G | A | 0.0839035 | 0.0178 | 2.43E-06 |  | 11,279 | 179,600 | -0.0212511 | 0.0169164 | 0.209027 |
| 10 | rs292441 | A | G | -0.0724954 | 0.0149 | 1.14E-06 |  | 11,279 | 179,600 | -0.0158723 | 0.0156521 | 0.31055 |
| 11 | rs35404050 | T | C | 0.0843044 | 0.0176 | 1.67E-06 |  | 11,279 | 179,600 | -0.0145808 | 0.0182017 | 0.423092 |
| 12 | rs45595836 | T | C | 0.138996 | 0.0272 | 3.22E-07 |  | 11,279 | 179,600 | -0.0193136 | 0.0321976 | 0.54861 |
| 13 | rs4750990 | C | T | 0.0680968 | 0.0141 | 1.37E-06 |  | 11,279 | 179,600 | 0.0121285 | 0.0141373 | 0.390943 |
| 14 | rs644552 | A | G | 0.159403 | 0.0346 | 4.08E-06 |  | 11,279 | 179,600 | -0.0324012 | 0.0341561 | 0.342814 |
| 15 | rs6692705 | G | A | -0.0656005 | 0.0141 | 3.28E-06 |  | 11,279 | 179,600 | -0.0468709 | 0.0144564 | 0.00118601 |
| 16 | rs76397219 | G | A | 0.140297 | 0.0303 | 3.65E-06 |  | 11,279 | 179,600 | 0.0209075 | 0.0337546 | 0.535656 |
| 17 | rs77691144 | C | T | 0.207406 | 0.0435 | 1.86E-06 |  | 11,279 | 179,600 | 0.106306 | 0.0650903 | 0.102426 |
| 18 | rs7783557 | C | T | -0.0670042 | 0.0146 | 4.45E-06 |  | 11,279 | 179,600 | 0.0013304 | 0.0148669 | 0.928695 |
| 19 | rs78058104 | A | G | 0.187898 | 0.0397 | 2.21E-06 |  | 11,279 | 179,600 | -0.00751465 | 0.0423485 | 0.859157 |
| 20 | rs78653484 | T | C | -0.176296 | 0.0385 | 4.67E-06 |  | 11,279 | 179,600 | -0.00491695 | 0.030052 | 0.870035 |
| 21 | rs78827416 | A | G | 0.130502 | 0.0266 | 9.29E-07 |  | 11,279 | 179,600 | -0.0371349 | 0.0229374 | 0.105454 |
| 22 | rs79940520 | G | A | 0.0953992 | 0.0207 | 4.05E-06 |  | 11,279 | 179,600 | 0.00910259 | 0.0220236 | 0.679379 |
| 23 | rs9366877 | G | A | -0.0684994 | 0.0139 | 8.31E-07 |  | 11,279 | 179,600 | -0.0250689 | 0.0145889 | 0.0857334 |
| 24 | rs9389208 | T | C | 0.0672006 | 0.0144 | 3.06E-06 |  | 11,279 | 179,600 | 0.00585618 | 0.0141916 | 0.679862 |

SNP, single nucleotide polymorphism; EA, effect allele; OA, other allele; SE, standard error; ASD, autism spectrum disorder; GDM, gestational diabetes mellitus.

**Supplementary Table S2.4** Information of identified SNPs in exposure (T1DM) and outcomes (ASD).

|  |  | | | **Exposure (T1DM)** | | |  | **Outcome (ASD)** | | | | |
| --- | --- | --- | --- | --- | --- | --- | --- | --- | --- | --- | --- | --- |
|  | **SNP** | **EA** | **OA** | **β** | **SE** | ***p* value** |  | **Case** | **Control** | **β** | **SE** | ***p* value** |
| 1 | rs115470510 | A | G | 1.40903 | 0.0460911 | 3.02E-205 |  | 18,382 | 27,969 | -0.00150113 | 0.036 | 0.965999941 |
| 2 | rs117557295 | C | A | -1.05621 | 0.117338 | 2.23E-19 |  | 18,382 | 27,969 | -0.0179971 | 0.0651 | 0.782699398 |
| 3 | rs12148472 | C | T | -0.216958 | 0.0395748 | 4.20E-08 |  | 18,382 | 27,969 | -0.0597018 | 0.0217 | 0.005918069 |
| 4 | rs12928537 | A | G | -0.16057 | 0.0293578 | 4.52E-08 |  | 18,382 | 27,969 | 0.0057037 | 0.0148 | 0.702300536 |
| 5 | rs146502328 | A | G | 0.591597 | 0.0634101 | 1.06E-20 |  | 18,382 | 27,969 | 0.0955011 | 0.0528 | 0.070610616 |
| 6 | rs146852891 | T | G | -0.745272 | 0.0903046 | 1.55E-16 |  | 18,382 | 27,969 | -0.00460057 | 0.054 | 0.932400075 |
| 7 | rs199618686 | G | A | -1.18908 | 0.121516 | 1.30E-22 |  | 18,382 | 27,969 | 0.0249999 | 0.0441 | 0.571099789 |
| 8 | rs2018705 | G | T | -0.186627 | 0.0323633 | 8.09E-09 |  | 18,382 | 27,969 | 0.00910129 | 0.0156 | 0.56150031 |
| 9 | rs2071463 | T | C | -0.766291 | 0.0418953 | 9.83E-75 |  | 18,382 | 27,969 | 0.001998 | 0.0189 | 0.913800097 |
| 10 | rs56994090 | C | T | -0.177446 | 0.0265483 | 2.33E-11 |  | 18,382 | 27,969 | -0.000899595 | 0.0146 | 0.95110004 |
| 11 | rs7130222 | G | T | -0.179019 | 0.0310595 | 8.23E-09 |  | 18,382 | 27,969 | -0.0320024 | 0.015 | 0.033269785 |
| 12 | rs74203920 | T | C | 0.377671 | 0.0634671 | 2.67E-09 |  | 18,382 | 27,969 | 0.00740253 | 0.0542 | 0.890900084 |
| 13 | rs876498 | A | G | 0.17432 | 0.0275611 | 2.53E-10 |  | 18,382 | 27,969 | -0.0172988 | 0.014 | 0.215799781 |
| 14 | rs9258224 | A | G | -0.51222 | 0.0629088 | 3.88E-16 |  | 18,382 | 27,969 | 0.0606999 | 0.0256 | 0.017869813 |
| 15 | rs9270523 | G | T | 0.98982 | 0.0283344 | 2.31E-267 |  | 18,382 | 27,969 | -0.0267004 | 0.0158 | 0.09224015 |

SNP, single nucleotide polymorphism; EA, effect allele; OA, other allele; SE, standard error; T1DM, type 1 diabetes mellitus; ASD, autism spectrum disorder.

**Supplementary Table S2.5** Information of identified SNPs in exposure (T2DM) and outcomes (ASD).

|  |  | | | **Exposure (T2DM)** | | |  | **Outcome (ASD)** | | | | |
| --- | --- | --- | --- | --- | --- | --- | --- | --- | --- | --- | --- | --- |
|  | **SNP** | **EA** | **OA** | **β** | **SE** | ***p* value** |  | **Case** | **Control** | **β** | **SE** | ***p* value** |
| 1 | rs10084393 | A | C | -0.061283 | 0.00820794 | 8.25E-14 |  | 18,382 | 27,969 | -0.0444954 | 0.0185 | 0.016230055 |
| 2 | rs10404726 | T | C | -0.0422001 | 0.00681874 | 6.06E-10 |  | 18,382 | 27,969 | 0.000499875 | 0.0141 | 0.974499902 |
| 3 | rs10408179 | C | T | -0.0567973 | 0.00685658 | 1.19E-16 |  | 18,382 | 27,969 | -0.0179971 | 0.014 | 0.196999897 |
| 4 | rs1046316 | G | A | 0.069643 | 0.00703756 | 4.34E-23 |  | 18,382 | 27,969 | 0.00830439 | 0.0146 | 0.570999858 |
| 5 | rs10466811 | A | G | -0.0512322 | 0.00808935 | 2.40E-10 |  | 18,382 | 27,969 | 0.0128965 | 0.0154 | 0.401700156 |
| 6 | rs10770143 | T | C | -0.0666259 | 0.00716832 | 1.48E-20 |  | 18,382 | 27,969 | 0.0044003 | 0.0153 | 0.774399386 |
| 7 | rs10802132 | T | C | 0.0404602 | 0.00724107 | 2.30E-08 |  | 18,382 | 27,969 | -0.0227979 | 0.015 | 0.128000009 |
| 8 | rs10882099 | C | T | -0.0691246 | 0.00682772 | 4.32E-24 |  | 18,382 | 27,969 | 0.000900405 | 0.014 | 0.95110004 |
| 9 | rs10885123 | T | C | -0.0591593 | 0.0101393 | 5.39E-09 |  | 18,382 | 27,969 | -0.0268985 | 0.0206 | 0.19219983 |
| 10 | rs10965247 | G | A | -0.107939 | 0.00964083 | 4.26E-29 |  | 18,382 | 27,969 | 0.0108992 | 0.0182 | 0.549300507 |
| 11 | rs10974438 | C | A | 0.0431823 | 0.00704162 | 8.65E-10 |  | 18,382 | 27,969 | -0.001998 | 0.0148 | 0.890999992 |
| 12 | rs11052566 | T | C | 0.0758584 | 0.0126018 | 1.75E-09 |  | 18,382 | 27,969 | 0.0511018 | 0.0284 | 0.071969751 |
| 13 | rs11169182 | T | C | 0.0417543 | 0.00683204 | 9.87E-10 |  | 18,382 | 27,969 | 0.00980181 | 0.0139 | 0.479399967 |
| 14 | rs112088479 | T | C | 0.069229 | 0.00988469 | 2.49E-12 |  | 18,382 | 27,969 | 0.0235993 | 0.0245 | 0.336300103 |
| 15 | rs11257659 | T | C | 0.0811613 | 0.00769491 | 5.22E-26 |  | 18,382 | 27,969 | -0.00729656 | 0.0166 | 0.658200185 |
| 16 | rs114322470 | G | T | -0.224936 | 0.02366 | 1.96E-21 |  | 18,382 | 27,969 | -0.0437968 | 0.0575 | 0.446600289 |
| 17 | rs11558471 | G | A | -0.0818036 | 0.00704937 | 3.91E-31 |  | 18,382 | 27,969 | 0.0243952 | 0.0147 | 0.097310566 |
| 18 | rs11631200 | A | G | 0.0423933 | 0.00684032 | 5.73E-10 |  | 18,382 | 27,969 | 0.0513963 | 0.014 | 0.000246899 |
| 19 | rs11720108 | T | C | -0.0761081 | 0.00902742 | 3.43E-17 |  | 18,382 | 27,969 | -0.010697 | 0.016 | 0.502400427 |
| 20 | rs1182436 | C | T | 0.0738976 | 0.00974199 | 3.31E-14 |  | 18,382 | 27,969 | 0.010697 | 0.019 | 0.572400494 |
| 21 | rs11899863 | T | C | -0.138821 | 0.0162393 | 1.25E-17 |  | 18,382 | 27,969 | -0.0320997 | 0.0234 | 0.170099877 |
| 22 | rs12001437 | C | T | 0.041023 | 0.00712404 | 8.49E-09 |  | 18,382 | 27,969 | -0.0134985 | 0.0144 | 0.348599693 |
| 23 | rs12195206 | T | C | 0.0501421 | 0.00798883 | 3.46E-10 |  | 18,382 | 27,969 | 0.00870203 | 0.0179 | 0.626100427 |
| 24 | rs12549902 | A | G | 0.0589031 | 0.00691348 | 1.60E-17 |  | 18,382 | 27,969 | -0.00599795 | 0.0141 | 0.672499553 |
| 25 | rs13067541 | T | C | -0.0743936 | 0.0129962 | 1.04E-08 |  | 18,382 | 27,969 | 0.0158044 | 0.024 | 0.510500298 |
| 26 | rs13427924 | C | T | -0.0400364 | 0.00681282 | 4.19E-09 |  | 18,382 | 27,969 | -0.001998 | 0.0139 | 0.887799917 |
| 27 | rs1397566 | G | A | -0.0416204 | 0.0068898 | 1.53E-09 |  | 18,382 | 27,969 | 0.016902 | 0.0141 | 0.229799987 |
| 28 | rs141433349 | A | G | 0.110395 | 0.0195216 | 1.56E-08 |  | 18,382 | 27,969 | 0.075803 | 0.0368 | 0.039190427 |
| 29 | rs142682088 | A | G | -0.110789 | 0.017457 | 2.20E-10 |  | 18,382 | 27,969 | 0.0164047 | 0.0367 | 0.654999548 |
| 30 | rs1604038 | T | C | -0.0538948 | 0.00774472 | 3.43E-12 |  | 18,382 | 27,969 | 0.00750179 | 0.0151 | 0.6190005 |
| 31 | rs1646629 | T | C | 0.0517495 | 0.00686434 | 4.74E-14 |  | 18,382 | 27,969 | 0.00819632 | 0.0142 | 0.562299892 |
| 32 | rs17036160 | T | C | -0.0918512 | 0.00916178 | 1.18E-23 |  | 18,382 | 27,969 | 0.0111971 | 0.0201 | 0.576600481 |
| 33 | rs17168486 | T | C | 0.0496089 | 0.00851563 | 5.69E-09 |  | 18,382 | 27,969 | -0.00399798 | 0.0181 | 0.824199981 |
| 34 | rs17383290 | G | A | -0.0484758 | 0.00773841 | 3.74E-10 |  | 18,382 | 27,969 | 0.013298 | 0.0139 | 0.339500173 |
| 35 | rs17596617 | T | C | 0.0402577 | 0.00731609 | 3.74E-08 |  | 18,382 | 27,969 | -0.0133994 | 0.015 | 0.370000235 |
| 36 | rs17772814 | A | G | -0.0804395 | 0.0116417 | 4.86E-12 |  | 18,382 | 27,969 | -0.0421979 | 0.0313 | 0.1778001 |
| 37 | rs17791633 | C | T | -0.0618274 | 0.0103666 | 2.46E-09 |  | 18,382 | 27,969 | 0.00260339 | 0.0258 | 0.919999942 |
| 38 | rs1925670 | G | T | -0.0390442 | 0.00684577 | 1.17E-08 |  | 18,382 | 27,969 | 0.00490199 | 0.0141 | 0.727799914 |
| 39 | rs1966 | T | C | 0.0483694 | 0.00813584 | 2.76E-09 |  | 18,382 | 27,969 | 0.00019998 | 0.0166 | 0.990599992 |
| 40 | rs1988138 | T | C | -0.046698 | 0.00800576 | 5.44E-09 |  | 18,382 | 27,969 | 0.0010994 | 0.0179 | 0.951799999 |
| 41 | rs2303700 | C | T | -0.0480432 | 0.00728101 | 4.16E-11 |  | 18,382 | 27,969 | -0.023697 | 0.0152 | 0.118799879 |
| 42 | rs2394576 | C | T | 0.0424454 | 0.00686602 | 6.33E-10 |  | 18,382 | 27,969 | 0.0377019 | 0.0144 | 0.0089 |
| 43 | rs2521247 | T | C | 0.0459196 | 0.00703788 | 6.82E-11 |  | 18,382 | 27,969 | 0.0204985 | 0.0165 | 0.214799863 |
| 44 | rs2682907 | A | G | -0.0602319 | 0.00694845 | 4.38E-18 |  | 18,382 | 27,969 | -0.00490199 | 0.0146 | 0.738999254 |
| 45 | rs28624681 | T | C | -0.076247 | 0.00755133 | 5.69E-24 |  | 18,382 | 27,969 | -0.0331949 | 0.0189 | 0.079330465 |
| 46 | rs2894553 | T | C | -0.0759144 | 0.0123906 | 8.97E-10 |  | 18,382 | 27,969 | -0.048004 | 0.0246 | 0.050790209 |
| 47 | rs3096301 | T | C | 0.0399613 | 0.00712506 | 2.04E-08 |  | 18,382 | 27,969 | 0.001998 | 0.0174 | 0.90829997 |
| 48 | rs348330 | A | G | -0.0490562 | 0.00711982 | 5.58E-12 |  | 18,382 | 27,969 | -0.017197 | 0.015 | 0.252199952 |
| 49 | rs34872471 | C | T | 0.254237 | 0.00830097 | 5.29E-206 |  | 18,382 | 27,969 | -0.0030952 | 0.0154 | 0.840499966 |
| 50 | rs35005436 | T | C | -0.0621371 | 0.0106304 | 5.06E-09 |  | 18,382 | 27,969 | -0.0159973 | 0.0201 | 0.42460001 |
| 51 | rs35120418 | A | C | 0.040337 | 0.00687763 | 4.49E-09 |  | 18,382 | 27,969 | 0.0140015 | 0.0142 | 0.324300038 |
| 52 | rs35352848 | C | T | -0.0705068 | 0.00741446 | 1.92E-21 |  | 18,382 | 27,969 | 0.00190181 | 0.017 | 0.911500057 |
| 53 | rs35529305 | A | G | -0.0734098 | 0.0131158 | 2.18E-08 |  | 18,382 | 27,969 | 0.0165031 | 0.0284 | 0.561000179 |
| 54 | rs35658696 | G | A | 0.106064 | 0.014359 | 1.51E-13 |  | 18,382 | 27,969 | 0.0806029 | 0.0318 | 0.011240095 |
| 55 | rs35824707 | G | A | 0.105366 | 0.0156771 | 1.80E-11 |  | 18,382 | 27,969 | 0.0411971 | 0.0347 | 0.234899991 |
| 56 | rs35895680 | A | C | -0.0419803 | 0.00742105 | 1.54E-08 |  | 18,382 | 27,969 | 0.00929665 | 0.0151 | 0.537300197 |
| 57 | rs3731503 | C | T | 0.0413463 | 0.00724711 | 1.16E-08 |  | 18,382 | 27,969 | 0.0194989 | 0.0155 | 0.207499952 |
| 58 | rs3736907 | A | C | -0.0439305 | 0.00772418 | 1.29E-08 |  | 18,382 | 27,969 | -0.00950503 | 0.0159 | 0.55240042 |
| 59 | rs3763432 | T | C | -0.0398479 | 0.00685305 | 6.08E-09 |  | 18,382 | 27,969 | 0.00990083 | 0.0149 | 0.50940007 |
| 60 | rs3887925 | T | C | 0.0483411 | 0.00684735 | 1.67E-12 |  | 18,382 | 27,969 | 0.0124028 | 0.014 | 0.374200187 |
| 61 | rs4240624 | A | G | -0.0608692 | 0.00968592 | 3.29E-10 |  | 18,382 | 27,969 | 0.00890027 | 0.024 | 0.709300066 |
| 62 | rs429150 | C | T | 0.0450307 | 0.00682391 | 4.14E-11 |  | 18,382 | 27,969 | 0.022399 | 0.0139 | 0.107700074 |
| 63 | rs4374177 | G | A | -0.0690122 | 0.0107479 | 1.35E-10 |  | 18,382 | 27,969 | 0.00329542 | 0.0185 | 0.859500038 |
| 64 | rs4862423 | T | C | 0.0452028 | 0.00701568 | 1.17E-10 |  | 18,382 | 27,969 | 0.00669752 | 0.0142 | 0.635800697 |
| 65 | rs488321 | C | T | -0.0861415 | 0.0115036 | 6.98E-14 |  | 18,382 | 27,969 | -0.0308007 | 0.0188 | 0.101600068 |
| 66 | rs4937325 | C | T | -0.0478799 | 0.007223 | 3.38E-11 |  | 18,382 | 27,969 | -0.0018982 | 0.0153 | 0.902699912 |
| 67 | rs4968896 | G | T | 0.0431248 | 0.00683265 | 2.76E-10 |  | 18,382 | 27,969 | 0.0222967 | 0.0139 | 0.109299959 |
| 68 | rs4981709 | G | A | 0.0390506 | 0.00693674 | 1.81E-08 |  | 18,382 | 27,969 | 0.0120017 | 0.0141 | 0.397499878 |
| 69 | rs498475 | A | G | -0.0702015 | 0.00711562 | 5.85E-23 |  | 18,382 | 27,969 | -0.00300451 | 0.0144 | 0.834099959 |
| 70 | rs56192168 | G | A | -0.0568257 | 0.00928492 | 9.34E-10 |  | 18,382 | 27,969 | 0.0101008 | 0.0211 | 0.632599727 |
| 71 | rs6030812 | A | G | -0.0514736 | 0.00942153 | 4.67E-08 |  | 18,382 | 27,969 | 0.0162965 | 0.0189 | 0.388399802 |
| 72 | rs6142129 | G | A | -0.0474735 | 0.00730934 | 8.31E-11 |  | 18,382 | 27,969 | 0.00300451 | 0.0147 | 0.840200044 |
| 73 | rs61779309 | C | T | 0.0629103 | 0.00861286 | 2.79E-13 |  | 18,382 | 27,969 | -0.0119977 | 0.0166 | 0.467799766 |
| 74 | rs618652 | G | T | -0.0418507 | 0.00682814 | 8.83E-10 |  | 18,382 | 27,969 | -0.00380276 | 0.0143 | 0.790800618 |
| 75 | rs62092443 | T | C | 0.0698079 | 0.0117407 | 2.75E-09 |  | 18,382 | 27,969 | -0.0164953 | 0.0307 | 0.59219945 |
| 76 | rs62319060 | C | T | 0.0429167 | 0.00686469 | 4.06E-10 |  | 18,382 | 27,969 | 0.00500249 | 0.0145 | 0.727699372 |
| 77 | rs66502159 | T | C | -0.112855 | 0.0117369 | 6.88E-22 |  | 18,382 | 27,969 | -0.028595 | 0.0222 | 0.196399781 |
| 78 | rs6850761 | C | A | -0.0454342 | 0.00709212 | 1.49E-10 |  | 18,382 | 27,969 | 0.0272993 | 0.0151 | 0.070740805 |
| 79 | rs6878122 | A | G | -0.0595912 | 0.00806625 | 1.49E-13 |  | 18,382 | 27,969 | 0.0204985 | 0.0153 | 0.181300082 |
| 80 | rs6931514 | G | A | 0.119851 | 0.00719289 | 2.46E-62 |  | 18,382 | 27,969 | -0.0071047 | 0.0155 | 0.646700207 |
| 81 | rs7018475 | G | T | 0.0989339 | 0.00753617 | 2.28E-39 |  | 18,382 | 27,969 | 0.00540458 | 0.016 | 0.735099284 |
| 82 | rs703980 | A | G | -0.048787 | 0.00680829 | 7.73E-13 |  | 18,382 | 27,969 | 0.0162965 | 0.0138 | 0.238099783 |
| 83 | rs7109575 | A | G | -0.0903571 | 0.00810131 | 6.89E-29 |  | 18,382 | 27,969 | -0.0212032 | 0.0182 | 0.243900112 |
| 84 | rs7226371 | G | A | 0.0574686 | 0.00893201 | 1.24E-10 |  | 18,382 | 27,969 | 0.0138049 | 0.0193 | 0.472899658 |
| 85 | rs72807614 | A | G | 0.0721667 | 0.00974009 | 1.27E-13 |  | 18,382 | 27,969 | 0.0592967 | 0.0289 | 0.040460384 |
| 86 | rs72932523 | A | G | -0.0518791 | 0.00925186 | 2.05E-08 |  | 18,382 | 27,969 | -0.0542978 | 0.0219 | 0.013080068 |
| 87 | rs73040004 | C | T | 0.0703653 | 0.00819527 | 9.00E-18 |  | 18,382 | 27,969 | 0.00500249 | 0.0174 | 0.773800489 |
| 88 | rs73061095 | G | T | 0.0956295 | 0.00732944 | 6.58E-39 |  | 18,382 | 27,969 | 0.00549507 | 0.0149 | 0.710799314 |
| 89 | rs73115426 | C | T | 0.199451 | 0.0223095 | 3.89E-19 |  | 18,382 | 27,969 | -0.00479847 | 0.0491 | 0.921899968 |
| 90 | rs73224247 | C | T | -0.0735272 | 0.0115306 | 1.81E-10 |  | 18,382 | 27,969 | -0.0204005 | 0.0268 | 0.447000491 |
| 91 | rs73347525 | G | A | -0.0523298 | 0.00832412 | 3.25E-10 |  | 18,382 | 27,969 | -0.0166014 | 0.0192 | 0.386699847 |
| 92 | rs74046911 | T | C | -0.206625 | 0.0134382 | 2.37E-53 |  | 18,382 | 27,969 | 0.031799 | 0.0346 | 0.358200345 |
| 93 | rs74563586 | A | G | -0.232637 | 0.0392902 | 3.20E-09 |  | 18,382 | 27,969 | -0.0403013 | 0.0615 | 0.512399857 |
| 94 | rs74628648 | T | C | -0.0784778 | 0.01288 | 1.11E-09 |  | 18,382 | 27,969 | -0.0198048 | 0.0305 | 0.516399722 |
| 95 | rs7563362 | G | A | 0.0657717 | 0.00972742 | 1.37E-11 |  | 18,382 | 27,969 | 0.0119005 | 0.0201 | 0.552499641 |
| 96 | rs7697171 | T | C | -0.0481932 | 0.00684438 | 1.90E-12 |  | 18,382 | 27,969 | -0.0401035 | 0.0138 | 0.00372898 |
| 97 | rs7788808 | C | T | 0.0576708 | 0.00795383 | 4.15E-13 |  | 18,382 | 27,969 | 0.010505 | 0.0167 | 0.5292002 |
| 98 | rs7845219 | C | T | -0.0408162 | 0.00680411 | 1.99E-09 |  | 18,382 | 27,969 | -0.0057037 | 0.0139 | 0.684399999 |
| 99 | rs78486128 | A | G | 0.0667625 | 0.0112443 | 2.89E-09 |  | 18,382 | 27,969 | -0.00540458 | 0.0343 | 0.874399901 |
| 100 | rs7935034 | C | T | -0.0412498 | 0.00745253 | 3.11E-08 |  | 18,382 | 27,969 | -0.00350385 | 0.0146 | 0.808799921 |
| 101 | rs7948351 | T | C | -0.0550677 | 0.00960472 | 9.84E-09 |  | 18,382 | 27,969 | 0.0349037 | 0.0203 | 0.085719573 |
| 102 | rs7995767 | C | T | -0.10831 | 0.0192221 | 1.75E-08 |  | 18,382 | 27,969 | 0.000300045 | 0.0291 | 0.992599998 |
| 103 | rs8071043 | C | T | 0.0702501 | 0.00734389 | 1.11E-21 |  | 18,382 | 27,969 | 0.00709511 | 0.0149 | 0.633500555 |
| 104 | rs8100204 | A | G | 0.0748815 | 0.00932867 | 9.99E-16 |  | 18,382 | 27,969 | 0.0304031 | 0.0193 | 0.116000003 |
| 105 | rs8126001 | T | C | -0.0377239 | 0.00687928 | 4.17E-08 |  | 18,382 | 27,969 | -0.0177974 | 0.0148 | 0.227800147 |
| 106 | rs853974 | C | T | -0.0505198 | 0.00762886 | 3.54E-11 |  | 18,382 | 27,969 | 0.00709511 | 0.0158 | 0.654500526 |
| 107 | rs878521 | A | G | 0.0688112 | 0.00835164 | 1.73E-16 |  | 18,382 | 27,969 | -0.0239034 | 0.0164 | 0.144800146 |
| 108 | rs897558 | G | A | 0.038199 | 0.00682545 | 2.19E-08 |  | 18,382 | 27,969 | 0.0107981 | 0.0139 | 0.436699807 |
| 109 | rs9350408 | T | C | -0.0424767 | 0.00683082 | 5.02E-10 |  | 18,382 | 27,969 | -0.0206007 | 0.014 | 0.141599917 |
| 110 | rs9410573 | C | T | -0.0410166 | 0.00692417 | 3.15E-09 |  | 18,382 | 27,969 | 0.0144032 | 0.0142 | 0.310000219 |
| 111 | rs9505086 | C | T | 0.0626466 | 0.00687167 | 7.75E-20 |  | 18,382 | 27,969 | 0.011597 | 0.0143 | 0.417399573 |
| 112 | rs960312 | A | G | 0.0439186 | 0.00788619 | 2.56E-08 |  | 18,382 | 27,969 | 0.0485997 | 0.0197 | 0.013369957 |
| 113 | rs9843558 | A | G | -0.0639821 | 0.0115121 | 2.73E-08 |  | 18,382 | 27,969 | 0.0194006 | 0.0217 | 0.372000051 |

SNP, single nucleotide polymorphism; EA, effect allele; OA, other allele; SE, standard error; T2DM, type 2 diabetes mellitus; ASD, autism spectrum disorder.

**Supplementary Table S2.6** Information of identified SNPs in exposure (GDM) and outcomes (ASD).

|  |  | | | **Exposure (GDM)** | | |  | **Outcome (ASD)** | | | | |
| --- | --- | --- | --- | --- | --- | --- | --- | --- | --- | --- | --- | --- |
|  | **SNP** | **EA** | **OA** | **β** | **SE** | ***p* value** |  | **Case** | **Control** | **β** | **SE** | ***p* value** |
| 1 | rs1055080 | A | G | -0.20224 | 0.042291 | 1.73E-06 |  | 18,382 | 27,969 | 0.0170046 | 0.037 | 0.646200069 |
| 2 | rs10758593 | A | G | 0.0674627 | 0.01408 | 1.66E-06 |  | 18,382 | 27,969 | -0.0136021 | 0.014 | 0.332200284 |
| 3 | rs13053342 | A | G | 0.0671075 | 0.0141629 | 2.16E-06 |  | 18,382 | 27,969 | -0.010697 | 0.0143 | 0.456000165 |
| 4 | rs1402837 | T | C | 0.088837 | 0.0184198 | 1.41E-06 |  | 18,382 | 27,969 | 0.0162965 | 0.0165 | 0.323100031 |
| 5 | rs2187240 | A | G | 0.0988775 | 0.0212562 | 3.29E-06 |  | 18,382 | 27,969 | 0.00980181 | 0.0241 | 0.684600166 |
| 6 | rs2237897 | T | C | -0.133555 | 0.026516 | 4.73E-07 |  | 18,382 | 27,969 | 0.0288012 | 0.0348 | 0.408100381 |
| 7 | rs2256965 | G | A | 0.0766284 | 0.0141651 | 6.31E-08 |  | 18,382 | 27,969 | 0.00530404 | 0.0142 | 0.707499276 |
| 8 | rs28624681 | T | C | -0.0780739 | 0.0156154 | 5.74E-07 |  | 18,382 | 27,969 | -0.0331949 | 0.0189 | 0.079330465 |
| 9 | rs58667885 | A | G | 0.249375 | 0.0532941 | 2.88E-06 |  | 18,382 | 27,969 | 0.0598997 | 0.0669 | 0.370700353 |
| 10 | rs6021276 | C | T | 0.0678764 | 0.0143128 | 2.11E-06 |  | 18,382 | 27,969 | -0.0020978 | 0.0148 | 0.885599984 |
| 11 | rs613937 | G | A | -0.0780174 | 0.0161994 | 1.46E-06 |  | 18,382 | 27,969 | -0.0240973 | 0.0178 | 0.174900077 |
| 12 | rs6562107 | C | T | -0.0880244 | 0.0189264 | 3.31E-06 |  | 18,382 | 27,969 | 0.0122953 | 0.0156 | 0.428399544 |
| 13 | rs7123869 | C | T | 0.295913 | 0.0458942 | 1.14E-10 |  | 18,382 | 27,969 | -0.043002 | 0.054 | 0.426399808 |
| 14 | rs7227997 | A | G | -0.0741786 | 0.0161689 | 4.48E-06 |  | 18,382 | 27,969 | -0.0248051 | 0.0166 | 0.133599859 |
| 15 | rs74829505 | A | G | -0.217832 | 0.0434287 | 5.28E-07 |  | 18,382 | 27,969 | 0.0553022 | 0.0438 | 0.206800222 |
| 16 | rs75394590 | G | T | -0.216968 | 0.0467293 | 3.43E-06 |  | 18,382 | 27,969 | 0.0598031 | 0.0452 | 0.18609984 |
| 17 | rs76349839 | C | A | -0.205991 | 0.040959 | 4.93E-07 |  | 18,382 | 27,969 | 0.0263953 | 0.0539 | 0.624099738 |
| 18 | rs7722200 | C | T | -0.144082 | 0.0152551 | 3.56E-21 |  | 18,382 | 27,969 | -0.00789872 | 0.0151 | 0.599299617 |
| 19 | rs77315096 | G | T | -0.255231 | 0.0552765 | 3.89E-06 |  | 18,382 | 27,969 | -0.0355988 | 0.0542 | 0.511699509 |
| 20 | rs7756992 | G | A | 0.102687 | 0.014676 | 2.62E-12 |  | 18,382 | 27,969 | -0.00519647 | 0.0154 | 0.73479975 |
| 21 | rs9275373 | A | G | 0.158059 | 0.0208377 | 3.32E-14 |  | 18,382 | 27,969 | -0.0034961 | 0.0207 | 0.866099923 |

SNP, single nucleotide polymorphism; EA, effect allele; OA, other allele; SE, standard error; GDM, gestational diabetes mellitus; ASD, autism spectrum disorder.

**Supplementary Table S3** The results of MR-Egger's intercept analysis.

| **Exposure** | **Outcome** | **Egger_intercept** | **SE** | ***p* value** |
| --- | --- | --- | --- | --- |
| ASD | T1DM | 0.004739329 | 0.02067119 | 0.82077542 |
| ASD | T2DM | 0.003933082 | 0.006056172 | 0.524259565 |
| ASD | GDM | 0.02202911 | 0.012307438 | 0.087244761 |
| T1DM | ASD | 0.007452816 | 0.010764995 | 0.50091667 |
| T2DM | ASD | 0.005567708 | 0.003976771 | 0.164284962 |
| GDM | ASD | 0.012196626 | 0.010952774 | 0.279355616 |

ASD, autism spectrum disorder; T1DM, type 1 diabetes mellitus; T2DM, type 2 diabetes mellitus; GDM, gestational diabetes mellitus.

**Supplementary Table S4** The results of heterogeneity analysis.

| **Exposure** | **Outcome** | **Method** | **Q** | **Q_df** | **Q_*p* val** |
| --- | --- | --- | --- | --- | --- |
| ASD | T1DM | MR Egger | 14.10847333 | 22 | 0.897584608 |
| ASD | T1DM | Inverse variance weighted | 14.16103908 | 23 | 0.922166737 |
| ASD | T2DM | MR Egger | 18.18094224 | 18 | 0.443793822 |
| ASD | T2DM | Inverse variance weighted | 18.60694598 | 19 | 0.482298261 |
| ASD | GDM | MR Egger | 28.00550154 | 22 | 0.175498958 |
| ASD | GDM | Inverse variance weighted | 32.08380237 | 23 | 0.098413113 |
| T1DM | ASD | MR Egger | 23.94185646 | 13 | 0.031669119 |
| T1DM | ASD | Inverse variance weighted | 24.82458586 | 14 | 0.036339136 |
| T2DM | ASD | MR Egger | 135.0871166 | 111 | 0.059785924 |
| T2DM | ASD | Inverse variance weighted | 137.4726348 | 112 | 0.051390833 |
| GDM | ASD | MR Egger | 16.09748246 | 19 | 0.650764243 |
| GDM | ASD | Inverse variance weighted | 17.33750957 | 20 | 0.63096149 |

ASD, autism spectrum disorder; T1DM, type 1 diabetes mellitus; T2DM, type 2 diabetes mellitus; GDM, gestational diabetes mellitus.

**Supplementary Table S5** Literature report of SNPs.

| **SNP** | **Report** | **Source** |
| --- | --- | --- |
| rs3887925 | The rs3887925 is located in intron 1 of the ST6GAL1 gene and is associated with a reduced risk of T2DM (OR 0.94, 95%CI 0.92-0.96). | DOI: 10.1002/ijc.34046 |
| rs11558471 | The probability of T2DM decreases with the presence of rs11558471. Specifically, the odds ratio for T2DM is 0.55 when comparing the GG versus AA genotype, with a 95% confidence interval of 0.32 to 0.96. | DOI: 10.1038/s10038-022-01067-4 |
| rs6931514 | The rs6931514 variant in the CDKAL1 gene is a significant genetic factor contributing to the increased susceptibility to T2D in the studied Chinese population. Subjects with the variant alleles of this SNP showed an increased risk for T2D susceptibility in a dose-response manner. | DOI: 10.1038/jhg.2012.25 |
| rs34872471 | The single nucleotide polymorphism rs34872471 was identified as a significant variant in a Genome-Wide Association Study focusing on Type 2 Diabetes Mellitus (T2DM) in the Lebanese population. This SNP is located in the TCF7L2 gene, which is known to play a role in T2DM susceptibility. | DOI: 10.1038/srep07351 |
| rs6878122 | The rs6878122 located in ZBED3-AS1 gene showed a significant statistical correlation with T2D in Pakistanis. | DOI: 10.1016/j.gene.2021.145563 |
| rs7018475 | The CDKN2B-rs7018475 variant, along with other identified genetic variants, is associated with an increased risk of periodontitis and T2DM associated with periodontitis as comorbidity in the studied Southeastern Brazilian population. | DOI: 10.1007/s00784-020-03717-3 |
| rs348330 | T2D risk allele G carrying ABCB10 rs348330 variant is associated with increased expression of ABCB10 in human β cells. | DOI: 10.1016/j.molmet.2021.101403 |
| rs4862423 | The rs4862423 is associated with fasting glucose or diabetes status. Furthermore, rs4862423 is associated with subclinical atherosclerosis and coincides with a DHS highly accessible in human heart. | DOI: 10.1194/jlr.M064592 |
| rs878521 | The GCK gene plays a critical role in glucose metabolism and islet function. The presence of rs878521 in the vicinity of this gene suggests a potential regulatory impact on GCK expression or function, which could influence the development or progression of T2D. | DOI: 10.1038/s41598-019-44076-8 |
| rs853974 | The rs853974 to be associated with liver transplant-free survival. | DOI: 10.1136/gutjnl-2016-313598 |
| rs13427924 | The rs13427924 was significantly associated with coronary artery disease (OR 0.87, *p* = 1.01×10^-3^). | DOI: 10.1089/dna.2017.3688 |
| rs4240624 | The A allele of rs4240624, located in the PPP1R3B gene, was associated with hepatic steatosis (OR 1.28, *p* = 0.03) | DOI: 10.1016/j.cgh.2013.02.011 |
| rs17036160 | It was observed that lower Body Mass Index (BMI) values before pregnancy and at birth were associated with women having the PPARG rs17036160 TT genotype. | DOI: 10.3390/jpm12020243 |

T2DM, type 2 diabetes mellitus.
